# ROS- and Radiation Source-Dependent Modulation of Leukocyte Adhesion to Primary Microvascular Endothelial Cells

**DOI:** 10.3390/cells11010072

**Published:** 2021-12-27

**Authors:** Denise Eckert, Felicitas Rapp, Ayele T. Tsedeke, Jessica Molendowska, Robert Lehn, Markus Langhans, Claudia Fournier, Franz Rödel, Stephanie Hehlgans

**Affiliations:** 1 GSI Helmholtz Center for Heavy Ion Research, Department of Biophysics, Planckstraße 1, 64291 Darmstadt, Germany; D.Eckert@gsi.de (D.E.); F.Rapp@gsi.de (F.R.); A.Tsedeke@gsi.de (A.T.T.); J.Molendowska@gsi.de (J.M.); C.Fournier@gsi.de (C.F.); 2 Ernst-Berl Institut für Technische und Makromolekulare Chemie, Technical University Darmstadt, Alarich-Weiss-Straße 8, 64287 Darmstadt, Germany; Robert.Lehn@tu-darmstadt.de; 3 Department of Membrane Dynamics, Technical University Darmstadt, Alarich-Weiss-Straße 8, 64287 Darmstadt, Germany; markus.langhans@tu-darmstadt.de; 4 Department of Radiotherapy and Oncology, University Hospital, Goethe University Frankfurt, Theodor-Stern-Kai 7, 60590 Frankfurt am Main, Germany; Franz.Roedel@kgu.de

**Keywords:** adhesion, endothelial cells, inflammation, leukocytes, low-dose irradiation, shear stress

## Abstract

Anti-inflammatory effects of low-dose irradiation often follow a non-linear dose–effect relationship. These characteristics were also described for the modulation of leukocyte adhesion to endothelial cells. Previous results further revealed a contribution of reactive oxygen species (ROS) and anti-oxidative factors to a reduced leukocyte adhesion. Here, we evaluated the expression of anti-oxidative enzymes and the transcription factor Nrf2 (Nuclear factor-erythroid-2-related factor 2), intracellular ROS content, and leukocyte adhesion in primary human microvascular endothelial cells (HMVEC) upon low-dose irradiation under physiological laminar shear stress or static conditions after irradiation with X-ray or Carbon (C)-ions (0–2 Gy). Laminar conditions contributed to increased mRNA expression of anti-oxidative factors and reduced ROS in HMVEC following a 0.1 Gy X-ray and 0.5 Gy C-ion exposure, corresponding to reduced leukocyte adhesion and expression of adhesion molecules. By contrast, mRNA expression of anti-oxidative markers and adhesion molecules, ROS, and leukocyte adhesion were not altered by irradiation under static conditions. In conclusion, irradiation of endothelial cells with low doses under physiological laminar conditions modulates the mRNA expression of key factors of the anti-oxidative system, the intracellular ROS contents of which contribute at least in part to leucocyte adhesion, dependent on the radiation source.

## 1. Introduction

The endothelium is highly involved in immunological events [1], modulating infections as well as chronic inflammatory diseases, such as atherosclerosis and aging [2,3,4,5]. During inflammation, endothelial cells (EC) are transiently activated to bind and recruit leukocytes from the blood stream into tissue [6,7]. A critical step in this process is the adhesion, or “trapping”, of leukocytes to the EC to enable subsequent extravasation. In order to bind leukocytes, EC express elevated levels of adhesion molecules like vascular cell adhesion molecule (VCAM)-1, intercellular adhesion molecule (ICAM)-1 or E-selectin on their surfaces [8]. Upon binding of leukocytes, signal transduction is initiated that enables subsequent diapedesis. The expression of adhesion molecules is tightly regulated amongst others, by reactive oxygen species (ROS), which are essential signaling molecules in the regulation of vascular homeostasis [9,10,11]. While oxygen is omnipresent in living organisms, the formation of ROS covers physiological processes. Excessive ROS production, however, generates harmful intermediates that result in DNA damage or metabolic stress, cellular dysfunction, and cell death [12,13]. Besides intrinsic processes, e.g., leakage from mitochondria, oxidative stress can be induced by external factors including drugs, heavy metals and ionizing radiation [14]. To counteract (excessive) ROS, cells have developed anti-inflammatory defense mechanisms. Part of this defense includes anti-oxidative enzymes, such as Catalase, Superoxide Dismutase (SOD), or Glutathione Peroxidase (GPx), that scavenge ROS. In brief, oxygen is converted into superoxide anion by single-electron transfer, which is dismutated into hydrogen peroxide by SOD. Hydrogen peroxide is converted into water and oxygen by Catalase and GPx [9]. On a molecular level, upregulation of anti-oxidative enzymes is mainly regulated by Nrf2 (Nuclear factor-erythroid-2-related factor 2), a transcription factor that, upon sensation of oxidative stress, is translocated into the nucleus and binds to anti-oxidative response elements (ARE) [12,15]. This process initiates the translation of anti-oxidative proteins to subsequently restore the redox balance of the cell [16].

Blood vessels are constantly exposed to physiological shear stress by the blood flow (“hemodynamic forces”) [17]. If this flow becomes too strong or turbulent, it may contribute to vascular damage like atherosclerosis [18,19]. On the other hand, it is well described that EC in vivo and in vitro change their cytoskeleton structure, gene expression profile or even lose their function if this shear stress is abolished [20,21]. In vitro, EC adapt to laminar flow by changing their microtubule and microfilament structure, which helps to reduce the forces of flow, and is thought to contribute to the enhanced trans-endothelial transport [22]. In addition, when cells are cultured in parallel-plate shear chambers under oscillatory shear stress, the production of superoxide ($O_{2}^{-}$) is increased [21]. We have previously shown that an in vitro system to mimic physiological shear stress by cultivating EC in a “flow chamber” is suitable to maintain a physiological/natural EC phenotype [23].

Chronic inflammatory musculoskeletal diseases are mainly treated with anti-inflammatory non-steroidal or steroidal drugs or biologicals [24,25] that, however, are associated with severe side effects, especially when used for longer periods [26]. For decades, the application of low dose radiation therapy (LD-RT) has been an alternative treatment option with more than 37,000 patients treated in Germany per year [27]. In contrast to high-dose radiation used in cancer therapy, known to initiate inflammation [28,29] and changes in endothelium (e.g., apoptosis, senescence, or atherosclerosis), LD-RT induces pain-relieving, anti-inflammatory effects in chronic inflammatory and degenerative diseases [30,31,32]. Further, patients undergoing LD-RT showed a reduction of movement restrictions, but the underlying mechanisms have not been fully investigated yet. Notably, two different types of ionizing radiation are used in LD-RT: sparsely ionizing X-rays or densely ionizing irradiation by particles. Classically, X-rays are applied in multiple sessions of 0.5 Gy or 1 Gy per fraction while patients are exposed to alpha (α-) particles from Radon decay in particular spas and Radon galleries. The dose received in Radon spas can be estimated to be 2 mSv per treatment regimen [33]. LD-RT has anti-inflammatory effects by modulating the immune system, e.g., a diminished respiratory burst in macrophages [32], an increasing amount of SOD production [34] and transforming growth factor (TGF)-β [35], an anti-inflammatory shift in the Th17/Treg-ratio [36] and, among other modulations of immune cells, a decrease in the activation marker CD69 [37]. Regarding the anti-inflammatory effect of LD-RT, activation of EC and modulation of leukocyte adhesion has been shown to play a central role [38,39,40].

In this study, we aimed to elucidate in more detail the mechanism(s) underlying the anti-inflammatory effects of low-dose irradiation in endothelium in dependence of physiological laminar shear stress and the radiation source, either with X-ray or with Carbon (C)-ion particle irradiation conferring a higher linear energy transfer (LET_H2O_) of 323 keV/µm using doses of 0.1 and 0.5 Gy. To this end, we analyzed leukocyte adhesion to primary microvascular endothelial cells, intracellular ROS production, and the gene expression of adhesion molecules and anti-oxidative enzymes and their common transcription factor Nrf2.

## 2. Materials and Methods

### 2.1. Cultivation of Human Primary Microvasular Endothelial Cells

Human primary microvascular endothelial cells (HMVEC) were purchased from Cell Applications Inc. (San Diego, CA, USA) and maintained in supplemented microvascular endothelial cell growth medium (HMVEC medium) (PELOBiotech GmbH, Martinsried, Germany), 1% penicillin-streptomycin, 1% L-glutamine (both Biochrom, Berlin, Germany) at 37 °C in a 5% CO_2_ incubator with 95% humidity. For experiments, cells were used between passage 4 and 6, while maintenance of the characteristic endothelial cell morphology was checked visually.

### 2.2. Setup of Physiological Laminar Shear Stress and Static Conditions

For RNA isolation, flow cytometric measurements, and ROS analysis under laminar conditions, 1 × 10^5^ HMVEC were plated in 35 mm cell culture dishes (Thermo Scientific Nunclon, Waltham, MA, USA) inside the growth area of a positioning device (Warner Instruments, Holliston, MA, USA; slotted, 6 mm × 24.5 mm bath). For adhesion assays and immunostaining of Nrf2 and DNA double-strand breaks (DSB), additional autoclaved glass cover slips (Carl Roth, Karlsruhe, Germany) were used as a substrate to facilitate subsequent microscopic analysis. Cells were incubated for 30 min to allow for attachment, and 1.5 mL of HMVEC medium was added. After 24 h, a laminar flow was initiated using a flow chamber setup as described in Erbeldinger et al. [23]. In brief, the cell culture dishes were connected with an insert (custom-made) with two channels for in- and outflow. Glass bottles with medium were connected to respective duplicates of each condition with silicone tubes (all inside the incubator), and medium flow was initiated by a peristaltic pump (Ismatec, Landsberg am Lech, Germany). Laminar flow was applied for 24 h to allow for the cells to adapt before they were subjected to irradiation and stimulation.

For static conditions, 2 × 10^5^ HMVEC were plated in 35 mm cell culture dishes. For adhesion assays and immunostaining of Nrf2 and DNA double-strand (DSB) breaks, cells were grown on autoclaved glass cover slips to facilitate subsequent microscopic analysis. Cells were placed in the incubator for 30 min to allow for attachment, and 1 mL of HMVEC medium was added. After 24 h, the medium was changed, and 24 h thereafter irradiation and stimulation were performed.

### 2.3. Irradiation Procedures and Stimulation with Tumor Necrosis Factor Alpha

For irradiation, all samples including non-irradiated (sham-treated) controls were transported to the irradiation location. Directly after irradiation, the HMVEC medium was changed with HMVEC medium containing 10 ng/mL tumor necrosis factor alpha (TNF-α) (Miltenyi Biotec, Bergisch Gladbach, Germany), except for negative controls, and immediately reconnected to the flow chamber (laminar conditions) or placed in the incubator (static conditions) for a further 24 h.

#### 2.3.1. X-Irradiation

Using an X-ray tube (General Electrics, München, Germany), irradiation was performed with 16 mA cathode current and 250 kV acceleration voltage at a dose rate of 2 Gy/min. Doses applied were 0.1, 0.5, 1, and 2 Gy.

#### 2.3.2. Carbon (C)-Ion Irradiation

At the UNILAC facility of GSI Helmholtz Centre for Heavy Ion Research (Darmstadt, Germany), C-ion irradiation was performed. An LET_H2O_ of 323 keV/µm and an energy of 4.08 MeV/u were used. Details of the irradiation facility are given in [41]. In contrast to X-irradiation, due to the LET_H2O_ of C-ion particles and the Poisson distribution of the ion traversals, low doses of C-ion irradiation do not hit all cell nuclei. The calculations of fluence (particles/cm^2^) (1), mean number of hits (*N*), based on fluence and mean nuclear area of HMVEC (~200 µm^2^) (2), and the fraction of cells without hit (*P*_0_) (3) were calculated according to equations as previously published [23] and summarized in Table 1:

(1) $$Fluence\;\left(particles/{\mathrm{cm}}^{2}\right)\;=\frac{\mathrm{Dose}\;\left(\mathrm{Gy}\right)\times \;10^{9}}{1.602\;\times {\;\mathrm{LET}}_{H2O}\frac{keV}{µm}}$$

Mean number of hits *N* = *Fluence* × *Nuclear area* (2)

(3) $$P_{0}=e^{-N}\;$$

### 2.4. Treatment with N-Acetyl-L-Cysteine, Hydrogen Peroxide and the Nrf2 Activator AI-1

N-acetyl-L-cysteine (NAC, Sigma-Aldrich, Taufkirchen, Germany), a commonly used ROS scavenger, was dissolved in phosphate-buffered saline (PBS; Thermo Fisher Scientific, Darmstadt, Germany; the pH was adjusted to 7.5) and added to the cells at a concentration of 5 mM 90 min before harvesting. For the stimulation of intracellular ROS, cells were treated with 1000 µM hydrogen peroxide (H_2_O_2_; Carl Roth) 30 min before harvesting. For activation of Nrf2, cells were treated for 24 h with AI-1 (Merck, Darmstadt, Germany) at a concentration of 100 µM. Dimethyl sulfoxide (DMSO; AppliChem, Darmstadt, Germany) served as a solvent control.

### 2.5. Measurement of Intracellular ROS

Intracellular ROS levels were measured with 2′,7′, -dichlorodihydrofluorescein-diacetate (H_2_DCFDA: DCF assay; Thermo Fisher Scientific) by flow cytometry as described in [42]. In brief, HMVEC were incubated 90 min in serum-free Microvascular Endothelial Cell Growth Medium supplemented with 2 µM DCF before harvesting. Next, HMVEC were detached with Trypsin/EDTA (Sigma-Aldrich) for 5 min, resuspended, pelleted (300× *g*, 5 min, 4 °C), and washed with PBS. The cell pellet was collected by centrifugation at 300× *g* for 5 min at 4 °C. The mean fluorescence was measured using a BD FACSCanto™ II Cell Analyzer (BD, Heidelberg, Germany) and analyzed by Flowing Software v. 2.5.1 (University of Turku, Turku, Finland). To eliminate unspecific background, the mean fluorescence from untreated cells was subtracted.

### 2.6. RNA Isolation and cDNA Synthesis

HMVEC cells were cultured under laminar or static conditions in 35 mm dishes, irradiated with 0, 0.1, 0.5, or 1 Gy X-ray or C-ions and stimulated with TNF-α. After 24 h, inserts were removed, washed with PBS, and RNA was isolated using a NucleoSpin miRNA Kit (Macherey-Nagel, Düren, Germany). RNA (500 ng) was reverse transcribed using M-MLV reverse transcriptase (200 U; Promega, Walldorf, Germany), random hexamer primers (5 µM; Thermo Fisher Scientific), and dNTPs (500 µM; Carl Roth) in a ProFlex™ PCR System (Thermo Fisher Scientific) with the settings: 25 °C for 10 min; 37 °C for 30 min; 42 °C for 30 min.

### 2.7. Quantification of Anti-Oxidative Enzyme, Nrf2, eNOS and Adhesion Molecule mRNA Expression by Quantitative Real-Time PCR

For quantification of the mRNA expression of SOD1, GPx1, Catalase, Nrf2 (gene name: *NFE2L2,* nuclear factor erythroid-derived 2-like 2), ICAM-1, VCAM-1, E-Selectin (gene name: *SELE*, Selectin E) and endothelial nitric oxide synthase (eNOS, gene name: *NOS3*), quantitative PCR (qPCR) was performed with the GoTaq Probe qPCR Master Mix (Promega) on a QuantStudio 5 Real-Time PCR System (Thermo Fisher Scientific) using primer and probes (Eurofins Genomics, Ebersberg, Germany) or TaqMan Assays (Thermo Fisher Scientific) depicted in Tables S1 and S2, respectively. All samples were run in duplicate and normalized to the expression of the housekeeping gene 60S ribosomal protein L37a (RPL37A). For SOD1, GPx1, Catalase, and Nrf2 expression measurements, the standard curve methodology was used. By this, pCR2.1 plasmids containing the individual fragments amplified with the corresponding forward and reverse primer (Table S1) were cloned (The Original TA Cloning Kit, Thermo Fisher Scientific) and amplified (NucleoBond Xtra Midi Plus EF, Macherey-Nagel) according to the manufacturer’s recommendations. The correct insertion of the individual fragment was confirmed by sequencing (Eurofins Genomics).

### 2.8. Adhesion Assay

#### 2.8.1. Isolation of Peripheral Blood Lymphocytes

The adhesion assay was performed as described in Erbeldinger et al. [23]. Peripheral blood lymphocytes (PBL) were isolated from blood donated by volunteers in BD Vacutainer^®^ blood collection tubes (BD) with an integrated gel barrier to enable blood separation. For this, the tubes were centrifuged at 1500× *g* for 20 min at room temperature (RT). After centrifugation, the plasma is present in the upper phase and the second phase consists of the peripheral blood mononuclear cells (PBMC), which were transferred to a new tube, washed with PBS + 2% fetal bovine serum superior (FBS; Sigma-Aldrich), and centrifuged at 300× *g* for 8 min. Next, red blood cell lysis buffer (RBC, 8.29 g/L NH_4_Cl, 1 g/L KHCO_3_, 37.2 mg/L Na_2_EDTA in aqua dest, pH 7.2) was added for 5 min at RT to remove erythrocytes. To stop the reaction, PBS + 2% FBS was added, followed by centrifugation at 300× *g* for 8 min to collect PBMC. PBMC were plated in X-VIVO medium (X-VIVO 15, Lonza, Basel, Switzerland), supplemented with 1% penicillin-streptomycin and 3% heat-inactivated, autologous plasma in T75 flasks (Nunclon, Thermo Scientific), and incubated for 90 min at 37 °C and 5% CO_2_ to enable the attachment of monocytes. Then, the supernatant containing PBL was centrifuged (300× *g*, 8 min), and PBL were resuspended in serum-free RPMI 1640 medium + L-glutamin; 1% HEPES;1% penicillin-streptomycin (all from Biochrom).

#### 2.8.2. Staining and Imaging

For visualization, PBL were incubated for 60 min at 37 °C with 25 µg cell tracker green (CMFDA, 5-chloromethylfluorescein diacetate, Thermo Fisher Scientific; dissolved in 0.5 mL DMSO) which is a live cell marker. By using a Coulter Counter (Z-series, Beckmann Coulter, Krefeld, Germany), the cell number was determined. PBL were collected by centrifugation (300× *g*, 10 min) and diluted in RPMI 1640 + L-glutamin; 1% HEPES; 1% penicillin-streptomycin; 10% heat-inactivated FBS at a concentration of 1 × 10^6^ cells/mL. For adhesion assays under laminar flow conditions, 2.4 × 10^7^ CMFDA-stained PBL were added to 60 mL medium, transferred to glass bottles connected to cell culture dishes containing a confluent monolayer of HMVEC and laminar flow was applied by a peristaltic pump. For static conditions, 2 × 10^6^ stained PBL were used per 35 mm dish. Adhesion was allowed for 30 min under both conditions. Next, cells were fixed with 4% formaldehyde (Carl Roth)/PBS for 15 min at RT. After pretreatment with PBS/Triton X-100 (Carl Roth, 0.3%) for 10 min, cells were stained with phalloidin-tetramethylrhodamine B isothiocyanate (phalloidin-TRITC; 0.4 µg/mL) and 4′,6-diamidino-2-phenylindol (DAPI) (1 µg/mL, both from Sigma-Aldrich) in PBS/Triton X-100 (0.3%) for 45 min. Subsequently, cells were washed three times with PBS and once with distilled water. Finally, they were mounted on slides with Vectashield mounting medium (Vector Laboratories, Peterborough, UK). At least 6 microscopic fields (10× magnification) were recorded per replicate using a hybrid fluorescence microscope Revolve 4M (VWR, Darmstadt, Germany). Counting of adherent PBL and HMVEC was performed using ImageJ (U.S. National Institutes of Health, Bethesda, MD, USA). For this, the multichannel image (blue, green, red) was split in separate channels. The green channel (CellTracker Green CMFDA) was used for counting the number of PBL and the blue channel (DAPI) for total number of nuclei. F-actin staining of HMVEC (phalloidin-TRITC; red channel) was applied to verify the correct morphology of endothelial cells and to assure that leukocytes are directly attached to HMVEC. The number of PBL was subtracted from the total number of DAPI-positive cells and the result was defined as the number of HMVEC in each microscopic field. For semi-automatic counting, the threshold was separately adapted for each channel first and for segmentation, the function “watershed” was applied. Subsequently, the number of particles was analyzed with parameters “size” set between 10 and infinity and “circularity” set to 0.5–1. Afterwards, the ratio between the number of PBL to total number of HMVEC was determined per microscopic field and mean values were calculated from all images. Mean values from technical replicates were determined and mean + standard error of the mean (SEM) was calculated (EXCEL software, Microsoft, Redmond, WA, USA).

### 2.9. Immunofluorescence Staining and Quantification of Nrf2 Translocation and γH2AX Foci Formation

#### 2.9.1. Staining and Imaging

For the analysis of Nrf2 nuclear translocation and DNA DSB repair, HMVEC were exposed to laminar shear stress or cultured under normal cell culture conditions. Cells were irradiated with 0.1 Gy or were treated with the Nrf2 activator AI-1 or its solvent DMSO and stimulated with TNF-α. After 24 h, cells were fixed for 15 min with 4% formaldehyde (Carl Roth)/PBS at RT. After pretreatment with PBS/Triton X-100 for 10 min, cells were blocked with 5% bovine serum albumin (BSA, Carl Roth)/PBS for 60 min at RT. Next, cells were incubated with primary antibodies (Nrf2f, clone EP1808Y, 1:150, #ab62352, Abcam, Cambridge, UK); anti-phospho-H2AX Ser139 antibody, clone JBW301, 1:1000, #05-636, Millipore, Schwalbach, Germany) in 5% BSA/PBS and stained with phalloidin-TRITC (0.133 µg/mL) for 60 min at RT in the dark. After three washing steps with PBS, cells were stained with secondary antibodies (Goat-anti-mouse AlexaFluor 633, 1:500 (Thermo Fisher Scientific); Goat-anti-rabbit AlexaFluor 488, 1:500 (Thermo Fisher Scientific)) in 5% BSA/PBS for 60 min at RT in the dark. Subsequently, cells were washed three times with PBS and cell nuclei were stained with DAPI (1 µg/mL/PBS) for 10 min at RT in the dark. Finally, cells were washed once with PBS and mounted on slides with Vectashield mounting medium. Microscopic pictures were taken on a confocal microscope (LEICA TCS SP8, Leica, Wetzlar, Germany). Using the navigator tool, 16 pictures were selected for z-scan recordings. Four focus points were determined and from there, ten 1 µm steps were recorded in up- and downwards direction (10 µm).

#### 2.9.2. Quantification of Translocation of Nrf2

Nuclear translocation of Nrf2 was quantified using ImageJ. Briefly, outlines of cells with respect to F-actin (phalloidin-TRITC) staining and outlines of nuclei based on DAPI staining were traced with a freehand tool. The IntDen (product of Area and Mean Gray Value) was measured from the channel of interest (Nrf2, green) in the whole cell and nuclei. Subsequently, the ratio of the IntDen of the nuclei to the IntDen of the whole cell was determined and 50 cells per condition were analyzed.

#### 2.9.3. Quantification of Foci

For quantification of γH2AX foci in 50 to 200 nuclei per experiment, the MatLab tool FoCo [43] was used in a slightly modified version. For analysis, images were first converted to an 8-Bit RGB image. The Huang method, implemented in the FoCo MatLab tool, was used for initial nuclei separation and a minimal radius of nuclei of ten pixels and four times dilation-erosion cycle was used for the creation of the secondary mask. For foci detection, a maximal radius of three pixels was set. The minimal intensity was set to be the threefold value of the mean intensity of the γH2AX signal in all nuclei in the image. After the FoCo analysis, nuclei were sorted into cell cycle phases by integrated DAPI intensity of the nucleus and only G1 nuclei were chosen to calculate the mean γH2AX foci number per nucleus.

### 2.10. Measurement of Adhesion Molecules and Hsp70 by Flow Cytometry

HMVEC, exposed to laminar shear stress or cultured under static conditions, were irradiated and stimulated with TNF-α. To evaluate the influence of Nrf2 activation on the protein expression of adhesion molecules, cells were treated with the Nrf2 activator AI-1 or DMSO as a control. After 24 h, cells were detached with citric saline buffer (0.135 M KCl, 0.015 M sodium citrate) and incubated with fluorochrome-conjugated cell surface antibodies specific for adhesion molecules (ICAM-1-APC, clone HA58, 1:10, #559771, BD; VCAM-1-PE, clone IE10, 1:10, #FAB5649P, Bio-Techne GmbH, Wiesbaden, Germany; E-Selectin-FITC, clone BBIG-E5, 1:10, #BBA21, Bio-Techne GmbH) for 30 min. After washing, cells were fixed and permeabilized (FIX&PERM^®^ Cell Fixation and Permeabilization Kit (Nordic-MUbio, Susteren, The Netherlands), and incubated with a PerCP-conjugated antibody directed against heat shock protein 70 (Hsp70; clone EP1007Y, 1:80, #ab223390, Abcam) for 30 min, followed by a washing step. Flow cytometry was applied (CytoFlex S, Beckman Coulter GmbH, Krefeld, Germany) and the mean fluorescence intensity (MFI) of each fluorochrome was calculated using CytExpert Software 2.4 (Beckman Coulter GmbH).

### 2.11. Evaluation of Apoptosis

HMVEC were subjected to laminar flow conditions and stained with DAPI 24 h after irradiation and TNF-α stimulation. Pictures were taken with a confocal microscope (Leica DMI 4000B, Leica) and nuclei with typical apoptotic morphology were counted relative to the number of all nuclei present in the microscopic field. For each experiment, at least 1300 nuclei from at least six randomly chosen microscopic fields were evaluated.

### 2.12. Statistical Analysis

Data and statistical analyses were performed with EXCEL software or GraphPad Prism 8 (GraphPad Software, Inc., La Jolla, CA, USA) and are displayed as means + SEM with GraphPad Software. For statistical analysis, the Shapiro–Wilk test was performed first to test for samples’ normal distribution. Subsequently, for samples consisting of more than two groups which passed the normality test, the one-way ANOVA followed by post-hoc Tukey multiple comparison test was applied. In the case of not normally distributed samples, the Kruskal–Wallis test, followed by Dunn’s test was used for comparison of more than two groups. The Mann–Whitney U test was performed for the comparison of two groups. A *p*-value < 0.05 was considered statistically significant.

## 3. Results

### 3.1. Low Dose X-Irradiation of Endothelial Cells Increases the mRNA Expression of Anti-Oxidative Factors and Decreases ROS and Leukocyte Adhesion under Laminar Conditions

**Figure 1:**
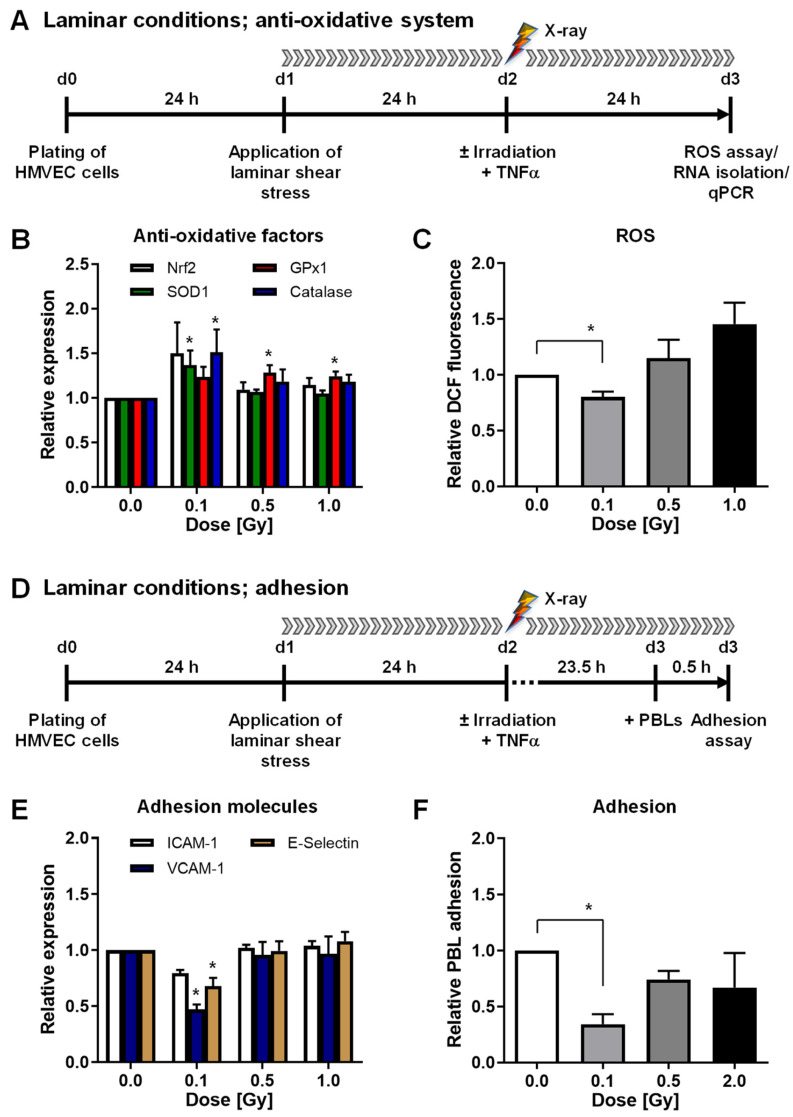
Low-dose X-irradiation modulates mRNA expression of anti-oxidative factors, reduces reactive oxygen species (ROS) and peripheral blood lymphocyte (PBL) adhesion to human microvascular endothelial cells (HMVEC) under laminar conditions. (**A**) Experimental setup: For analysis of the mRNA expression of anti-oxidative factors, ROS and adhesion molecules under shear stress, laminar flow conditions were applied 24 h after plating of HMVEC. (**B**) After irradiation and stimulation with tumor necrosis factor alpha (TNF-α), RNA was isolated to measure the mRNA expression of anti-oxidative factors (Nrf2, Nuclear factor-erythroid-2-related factor 2; SOD1, Superoxide Dismutase 1; GPx1, Glutathione Peroxidase 1; Catalase) by quantitative PCR (qPCR) (mean + SEM; *n* = 6) or (**C**) cells were subjected to analysis of ROS by flow cytometry (mean + SEM; *n* = 8–12). (**D**) For adhesion assays, PBL were added 23.5 h after irradiation and adhesion was allowed for 0.5 h under constant laminar flow. (**E**) Measurement of adhesion molecules vascular cellular adhesion molecule (VCAM)-1, intercellular adhesion molecule (ICAM)-1, and E-selectin expression was performed by qPCR (mean + SEM; *n* = 6). (**F**) For adhesion assays, HMVEC were plated on glass cover slips, PBL were stained with CellTracker Green CMFDA (5-chloromethylfluorescein diacetate) and added 23.5 h after irradiation for 30 min to the medium reservoir under permanent laminar flow. HMVEC cells and adhered PBL were fixed and stained with 4′,6-diamidino-2-dhenylindol (DAPI) and phalloidin-tetramethylrhodamine B isothiocyanate (phalloidin-TRITC). PBL adhesion was evaluated by microscopic counting of PBL relative to the number of endothelial cells (mean + SEM; *n* = 5–9). * *p* < 0.05, Kruskal-Wallis test vs. 0 Gy.

First, we aimed to examine the impact of low doses of X-rays (0.1 and 0.5 Gy) on the mRNA expression of the transcription factor Nrf2 and the anti-oxidative enzymes SOD1, GPx1, and Catalase 24 h after pro-inflammatory stimulation with TNF-α under more physiological laminar flow shear stress conditions. High doses of 1 or 2 Gy were used as a reference. In addition, mRNA expression of adhesion molecules ICAM-1, VCAM-1, and E-selectin and relative adhesion of PBL to HMVEC were analyzed. The time courses of the experiments are depicted in Figure 1A,D.

**Figure 2:**
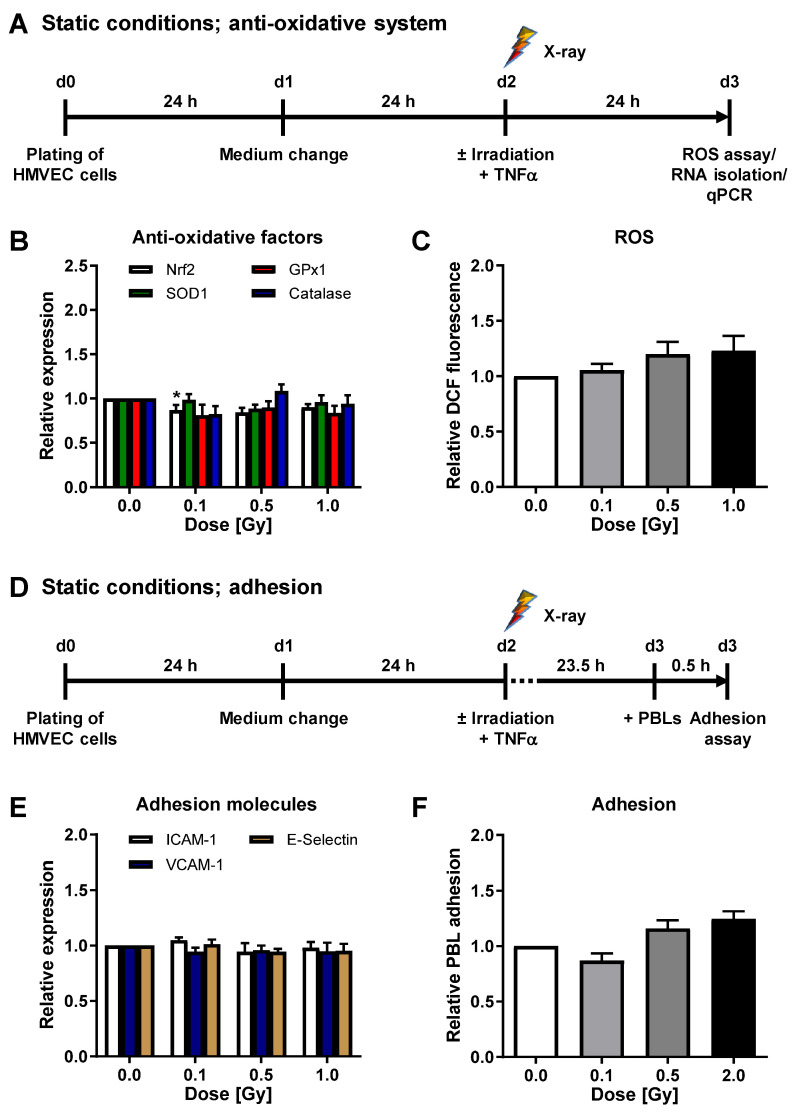
X-irradiation under static conditions does not modulate the mRNA expression of anti-oxidative enzymes in HMVEC cells nor PBL adhesion. (**A**) HMVEC were plated and incubated for 24 h. Medium was changed 24 h before irradiation and TNF-α treatment and ROS measurement and RNA isolation were performed at 24 h thereafter. (**B**) mRNA expression of anti-oxidative factors was evaluated by qPCR (mean + SEM; *n* = 5–6 * *p* < 0.05; Kruskal-Wallis test vs. 0 Gy). (**C**) Intracellular ROS were measured by flow cytometry (mean + SEM; *n* = 12). (**D**) Schematic setup of adhesion experiments. (**E**) mRNA expression of indicated adhesion molecules was measured by qPCR (mean + SEM; *n* = 6). (**F**) Microscopic evaluation of PBL adhesion was performed after fixation and DAPI and phalloidin-TRITC staining and values, relative to non-irradiated samples are shown (mean + SEM; *n* = 9).

Irradiation with 0.1 Gy induced the mRNA expression of factors Nrf2, SOD1, GPx1, and Catalase (Figure 1B). Concurrently, intracellular ROS, adhesion molecule mRNA expression, and PBL adhesion were significantly decreased after a 0.1 Gy exposure (Figure 1C,E,F and Figure S1A,B). To further analyze whether X-ray irradiation impacts the anti-oxidative system of HMVEC, we then applied the experimental settings depicted in Figure 2A,D. In contrast to laminar shear stress conditions, irradiation under static conditions did not modulate the mRNA expression of SOD1, GPx1, or Catalase (Figure 2B) nor the intracellular ROS content of HMVEC (Figure 2C and Figure S1C). Moreover, the mRNA expression of adhesion molecules ICAM-1, VCAM-1, and E-Selectin and PBL adhesion to HMVEC were not changed after static conditions (Figure 2E,F and Figure S1D).

**Figure 3:**
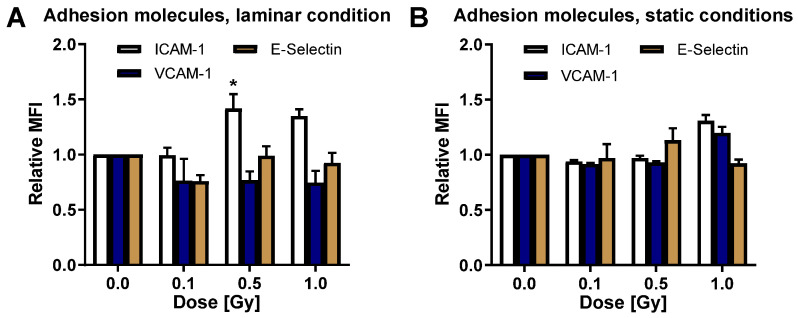
X-irradiation under laminar conditions reduces adhesion molecule surface expression. (**A**) For analysis of adhesion molecule protein expression under laminar conditions, HMVEC were exposed to laminar shear stress for 24 h, followed by irradiation and TNF-α stimulation. At 24 h after irradiation, cells were collected for flow cytometric analysis of ICAM-1, VCAM-1 and E-Selectin surface expression. Mean fluorescence intensities (MFI) + SEM are shown relative to non-irradiated cells. *n* = 4; * *p* < 0.05 (Mann-Whitney U test vs. +TNF-α). (**B**) For analysis of adhesion molecule protein expression under static conditions, HMVEC were plated, medium was changed and irradiation and TNF-α was applied. Adhesion molecule protein expression on the cell surface was measured by flow cytometry, 24 h after irradiation, and displayed as MFI relative to non-irradiated cells + SEM (*n* = 4).

To verify our findings on mRNA expression level, we measured the cell surface protein expression of cell adhesion molecules. Irradiation under laminar conditions resulted in a slightly reduced surface expression of VCAM-1 and E-Selectin after 0.1 Gy irradiation (Figure 3A). In concordance with the above-described findings on mRNA level, irradiation under static conditions did not modulate adhesion molecule protein expression (Figure 3B), confirming our previous findings [23].

To exclude that regulation of stress factors endothelial nitric oxide synthase (eNOS) or heat shock protein 70 (Hsp70) impacts the reduced adhesion upon 0.1 Gy, we measured eNOS mRNA and Hsp70 protein expression under the same conditions as described above. X-ray irradiation did neither modulate eNOS nor Hsp70 expression under shear stress (Figure S2A–C), nor under conventional static conditions (Figure S2D–F).

To elucidate whether DNA repair is involved in the modulations of anti-oxidative factors and adhesion processes observed following a 0.1 Gy exposure, we analyzed γH2Ax foci formation 24 h after X-ray irradiation (Figure S3A–C). X-ray irradiation with 0.1 Gy did not significantly enhance DNA damage. Further, cell death, as measured by the amount of apoptotic cells, was not significantly increased by X-ray irradiation with 0.1 Gy under shear stress (Figure S3D,E) with very low values of around 0.12% for 0 Gy and 0.1 Gy.

### 3.2. C-Ion Irradiation Modulates the Anti-Oxidative System of HMVEC and Decreases ROS and Leukocyte Adhesion under Laminar Conditions

**Figure 4:**
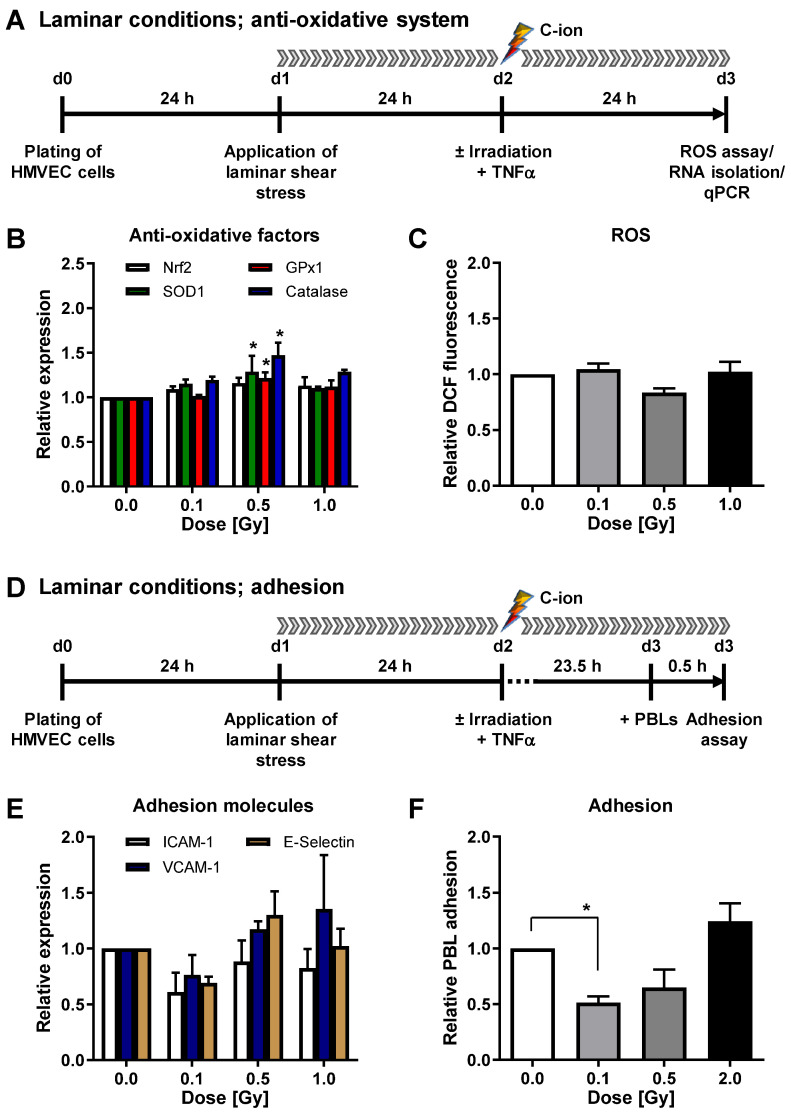
Carbon ion irradiation modulates the anti-oxidative defense of HMVEC cells and leukocyte adhesion. (**A**) Experimental settings: At 24 h after plating, HMVEC cells were exposed to laminar shear stress conditions. Irradiation with Carbon (C)-ions and TNF-α stimulation was applied. After 24 h, cells were collected, followed by ROS analysis and RNA isolation. (**B**) mRNA expression of anti-oxidative factors was measured by qPCR and values were displayed relative to non-irradiated cells (mean + SEM; *n* = 4; * *p* < 0.05, Kruskal-Wallis test vs. 0 Gy). (**C**) ROS analysis was performed by flow cytometry (mean + SEM; *n* = 4). (**D**) For adhesion experiments, PBL were added 23.5 h after irradiation to the flow chamber and adhesion was subsequently measured by microscopic evaluation. (**E**) mRNA expression of indicated adhesion molecules relative to non-irradiated cells is shown (mean + SEM; *n* = 3–4). (**F**) Adhesion of PBL was calculated relative to non-irradiated samples (mean + SEM; *n* = 6; * *p* < 0.05, one-way ANOVA vs. 0 Gy).

To further explore the role of ROS and anti-oxidative cellular mechanisms for PBL adhesion upon irradiation with high LET, we next applied C-ion particle irradiation using comparable doses and experimental setups as described before and depicted in Figure 4A,D. C-ion irradiation under laminar conditions resulted in an increase of Catalase mRNA expression at 0.1, 0.5, and 1 Gy, most pronounced at 0.5 Gy, and a significant upregulation of SOD1 and GPx1 upon 0.5 Gy C-ion exposure (Figure 4B), corresponding to a not significantly reduced ROS content 24 h after 0.5 Gy C-ion exposure (Figure 4C and Figure S4A).

The investigation of adhesion revealed a reduction of ICAM-1, VCAM-1, and E-Selectin adhesion molecule mRNA expression after a 0.1 Gy C-ion exposure (Figure 4E), a significantly reduced PBL adhesion after irradiation of HMVEC with 0.1 Gy, and a non-significant reduction of PBL adhesion at 0.5 Gy (Figure 4F and Figure S4B).

**Figure 5:**
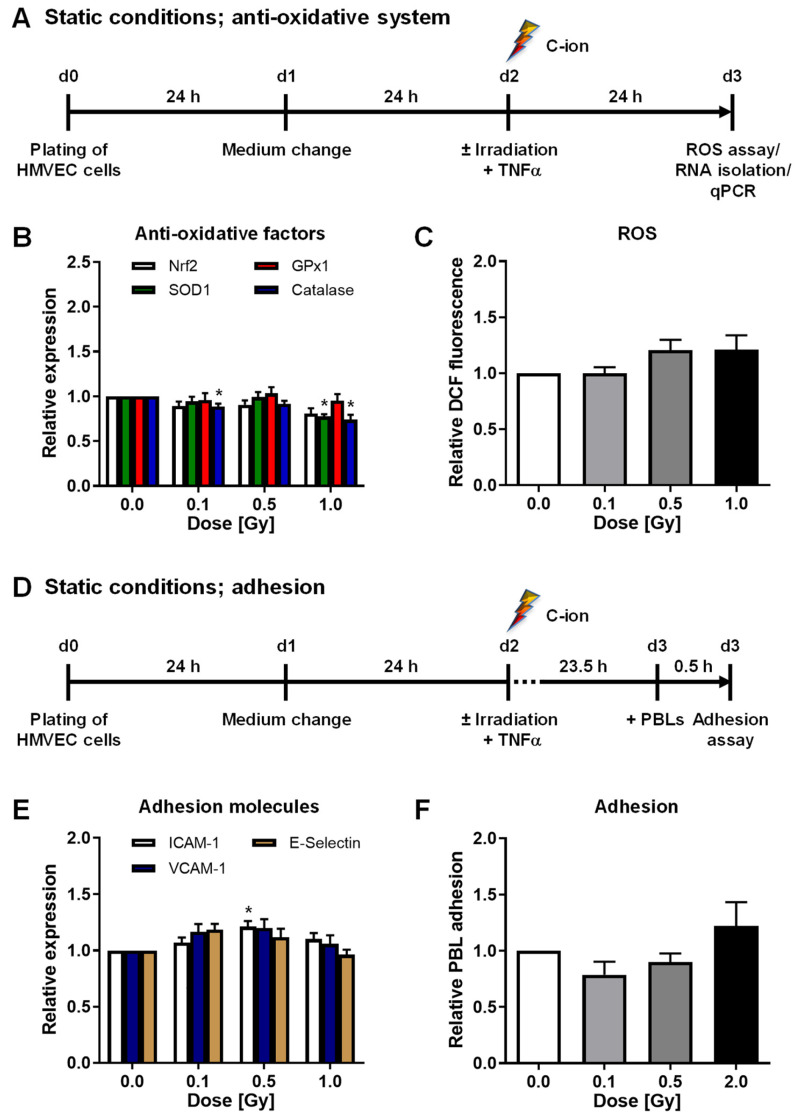
C-ion irradiation moderately changes the mRNA expression of anti-oxidative factors, ROS and adhesion of HMVEC cells under static conditions. (**A**) Experimental time course of analysis of C-ion irradiation-induced changes of the anti-oxidative defense of HMVEC, cultured under normal static conditions. (**B**) Relative mRNA expression of Nrf2, SOD1, GPx1 and Catalase at 24 h after C-ion irradiation (mean + SEM; *n* = 8; * *p* < 0.05, Kruskal-Wallis test vs. 0 Gy). (**C**) Intracellular ROS content, relative to non-irradiated cells, after C-ion irradiation of HMVEC with indicated doses (mean + SEM; *n* = 7–8). (**D**) Experimental setup of adhesion assays, performed 24 h after C-ion exposure. (**E**) Relative mRNA expression of indicated adhesion molecules after irradiation with C-ions (mean + SEM; *n* = 8; * *p* < 0.05, Kruskal-Wallis test vs. 0 Gy). (**F**) Relative PBL adhesion at 24 h after irradiation of HMVEC cells with C-ions (mean + SEM; *n* = 2–6).

By contrast, C-ion exposure of HMVEC cells cultured under conventional static conditions (experimental setup depicted in Figure 5A,D) slightly modulated the mRNA expression of anti-oxidative factors (Figure 5B) and adhesion molecules (Figure 5E), while intracellular ROS content (Figure 5C and Figure S4C) or PBL adhesion (Figure 5F and Figure S4D) were not affected at 24 h after irradiation.

Similar to X-ray irradiation, C-ion exposure did not modulate eNOS mRNA expression under shear stress (Figure S5A) or under static conditions (Figure S5B). Moreover, apoptosis did not contribute to the effects observed with very low levels up to 0.15% (Figure S6A,B).

### 3.3. Oxidative Stress Induces Leukocyte Adhesion to Primary HMVEC and Activation of Nrf2 Reduces Cellular Adhesion Molecules on the Surface of HMVEC

**Figure 6:**
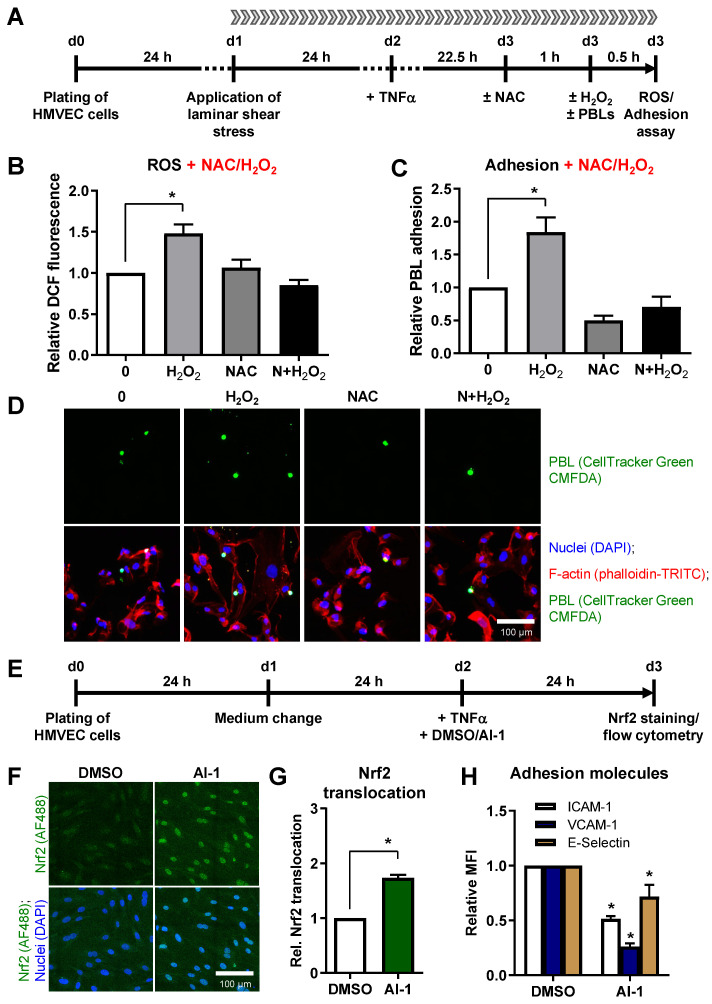
Oxidative stress induces leukocyte adhesion to HMVEC under shear stress and Nrf2 activation decreases adhesion molecule surface expression. (**A**) Experimental scheme: HMVEC were plated and laminar shear stress was applied 24 h before TNF-α stimulation. After 22.5 h, cells were either treated with the ROS scavenger N-acetyl-L-cysteine (NAC) or left untreated. Oxidative stress was induced 1 h later by addition of hydrogen peroxide either without (H_2_O_2_) or with pretreatment with NAC (N+H_2_O_2_). (**B**) ROS measurement was subsequently acquired by flow cytometry (Mean + SEM; *n* = 6; * *p* < 0.05, one-way ANOVA vs. 0 Gy). (**C**) For adhesion experiments, Cell Tracker Green-stained PBL were added to the medium reservoir of the flow chamber and adhesion to HMVEC was allowed for 30 min under laminar flow (Mean + SEM; *n* = 6; * *p* < 0.05, one-way ANOVA vs. 0 Gy). (**D**) Exemplary pictures, depicting adhered PBL (CellTracker Green CMFDA (5-chloromethylfluorescein diacetate)), either alone or merged with nuclei (DAPI, blue) and F-actin staining of HMVEC cells (phalloidin-TRITC, red). Bar, 100 µm. (**E**) To analyze the impact of Nrf2 activation on adhesion molecule expression, HMVEC were plated, medium was changed after 24 h and cells were treated with TNF-α and DMSO or the Nrf2 activator AI-1. After 24 h, cells were fixed, stained for Nrf2 (AF488, green) and DAPI (blue). (**F**) Depicted are exemplary pictures, showing Nrf2 staining alone (green channel) or merged with nuclei (DAPI, blue). (**G**) Samples were evaluated microscopically for Nrf2 translocation, or (**H**) adhesion molecules ICAM-1, VCAM-1 and E-Selectin were measured by flow cytometry and values calculated as MFI + SEM relative to DMSO-treated cells. *n* = 4; * *p* < 0.05 (Mann-Whitney U test vs. DMSO).

To investigate whether oxidative stress, provoked in HMVEC, impacts on PBL adhesion, we applied 1 mM hydrogen peroxide (H_2_O_2_) directly to the medium reservoir of the flow chamber at 23.5 h after TNF-α stimulation, incubated the cells for 30 min and measured the cellular ROS level. For adhesion assays, PBL were added concomitantly with H_2_O_2_ and adhesion was evaluated 30 min later (scheme depicted in Figure 6A).

Addition of H_2_O_2_ significantly increased DCF fluorescence by 48% (Figure 6B) and PBL adhesion by 85% (Figure 6C,D). These findings were confirmed by pretreatment of HMVEC with 5 mM NAC, a ROS scavenger, for 1 h, preventing enhanced ROS and PBL adhesion (Figure 6B–D).

Notably, treatment of HMVEC with H_2_O_2_ and NAC under static cell culture conditions yielded similar results as under physiological flow (Figure S7A), showing an induction of ROS (48%) and PBL adhesion (74%). These effects were abrogated when NAC pretreatment was applied (Figure S7B–D).

To analyze the direct impact of Nrf2 activation on adhesion molecule surface expression, we treated TNF-α stimulated HMVEC for 24 h with the Nrf2 activator AI-1 (Figure 6E), confirmed the nuclear translocation of Nrf2 by immunofluorescence staining (Figure 6F,G), and measured the surface expression of ICAM-1, VCAM-1 and E-Selectin by flow cytometry (Figure 6H). Importantly, Nrf2 activation significantly decreased ICAM-1, VCAM-1, and E-Selectin surface expression (Figure 6H).

In all static settings, controls without TNF-α treatment were measured for all conditions and compared with TNF-α stimulated but non-irradiated (0 Gy) HMVEC (Figure S8A). TNF-α significantly increased intracellular ROS levels (Figure S8B), decreased Nrf2, SOD1, and GPx1 mRNA expression, increased Catalase mRNA expression (Figure S8C), and induced mRNA and protein expression of adhesion molecules ICAM-1, VCAM-1, and E-selectin (Figure S8D,E) 24 h after stimulation. This resulted in an activation of HMVEC, shown by the 4.4 times increased PBL adhesion (Figure S8F). By contrast, mRNA expression of eNOS was significantly reduced after TNF-α stimulation (Figure S8G), while Hsp70 protein expression was enhanced (Figure S8H).

## 4. Discussion

LD-RT with photons or Radon exposure for the treatment of chronic inflammatory/degenerative musculoskeletal diseases exerts beneficial effects for patients, including pain relief and improvement of the quality of life by the modulation of inflammatory processes. Recently, these anti-inflammatory effects have been the subject of in vitro, in vivo, and patient studies, revealing a regulation of inflammatory pathways by affecting the activity of endothelial cells, macrophages, and leukocytes [38,40,44]. However, effects on immune cells exerted by charged particles, especially in a low dose range, are barely known.

While photon LD-RT is a common approach in a multitude of radiation therapy facilities [45], exposure to low doses of Radon is restricted to a few locations recently reviewed in [33]. Historically, the noble gas is delivered via inhalation in Radon galleries or by Radon containing bathes. For technical reasons, it is not possible to expose cell cultures directly to Radon, especially not when applying laminar flow to the cells, because Radon alike alpha-emitters are easily shielded by very thin layers of plastic. In addition, Radon does not emit a uniform radiation, but undergoes a series of radioactive decays, where alpha particles as well as β- and γ-radiation are emitted until stable lead (^208^ or ^206^Pb) is reached [33]. The most prominent alpha particle emitters in the Radon decay chain are ^222^Rn, ^218^Po and ^214^Po which emit alpha particle radiation with LET_H2O_ of 85.5, 80.2 and 67.1 keV/µm, respectively (personal communication Dr. A. Maier) as calculated with the software SRIM (stopping and range of ions in matter) described by Ziegler et al. [46]. In the present study, we used photon irradiation (X-rays) and compared it to different particle irradiation types to unravel potential differences. In former analyses, we used He-ions with an energy of 1.62 MeV/u and an LET_H2O_ of 76 keV/µm as a substitute for Radon and observed a decreased PBL adhesion to HMVEC after irradiation with a dose of 0.1 Gy [23]. In this study, we applied C-ions with an energy of 4.08 MeV/u and an LET_H2O_ of 323 keV/µm to further analyze heavy ion irradiation. For X-rays, we confirmed our former findings that PBL adhesion is reduced at 0.1 Gy when laminar flow is applied, while for C-ions, we showed for the first time that PBL adhesion is also significantly reduced after irradiation with 0.1 Gy (Figure 4F) similar to the findings for He-ion treatment. Interestingly, the ROS regulation is shifted with a most pronounced reduction following a 0.5 Gy exposure (Figure 4C).

On the basis of these findings, in the present study, we expanded our analyses to the impact of ROS and anti-oxidative defense mechanisms in primary endothelial cells (HMVEC) by analyzing anti-oxidative enzymes SOD1, GPx1, and Catalase and their common transcription factor Nrf2 after irradiation with low doses of X-ray and C-ions. Under physiological conditions using laminar flow, X-irradiation with 0.1 Gy induced the mRNA expression of anti-oxidative factors in inflammatory (TNF-α) stimulated HMVEC, reduced ROS levels, the expression of adhesion molecules ICAM-1, VCAM-1, and E-Selectin, and subsequent PBL adhesion (Figure 1B,C,E,F and Figure 3A). Exposure of stimulated HMVEC cells to low doses of C-ion particles, also under physiological flow, further resulted in a shift of the local maximum of the mRNA expression of anti-oxidative factors to 0.5 Gy, concomitant with a slightly decreased ROS detection at 0.5 Gy and a decreased mRNA expression of adhesion molecules at 0.1 Gy and PBL adhesion most pronounced at 0.1 and slightly at 0.5 Gy (Figure 4B,C,E,F). Similar to photon irradiation, C-ion irradiation of shear stress-exposed HMVEC thus seems to modulate the anti-oxidative response, but with a shift of the local maximum of anti-oxidative defense molecules and a local minimum of ROS to 0.5 Gy, while the process of PBL adhesion was most effectively hampered after a 0.1 Gy exposure. For that reason, we speculated that additional mechanisms might contribute to the decreased adhesion at 0.1 Gy. Since NO-dependent mechanisms are reported to impact endothelial inflammatory processes, we also analyzed eNOS mRNA expression in HMVEC exposed to C-ion irradiation at 0.1 Gy. However, eNOS mRNA expression was not modulated after low doses of C-ions, similar to X-ray irradiation. Thus, these findings do not support a role of NO in the reduced adhesion after LD-RT. Moreover, 0.1 Gy C-ion irradiation does not hit all cells (Table 1), and might therefore also be influenced by non-targeted effects, which we propose to further characterize in future experiments.

While the results for mRNA expression of adhesion molecules and PBL adhesion show a clear impact of LD-RT under laminar flow (Figure 1E,F and Figure 4E,F), the change in surface expression of CAMs is less obvious, as measured by flow cytometry (Figure 3A). This is probably due to a conformation change to dimers to form functioning, PBL binding integrin structures [47,48], which cannot be assessed by measuring a sole surface expression.

Oxidative stress covers a major cause of endothelial dysfunction contributing to inflammatory processes [9,49,50]. In our experiments, we observed an increase of ROS when non-irradiated HMVEC were stimulated with the proinflammatory cytokine TNF-α. In addition, SOD1, GPx1, and Nrf2 mRNA expression were slightly decreased while Catalase expression was increased (Figure S8B,C). Simultaneously, the expression of adhesion molecules ICAM-1, VCAM-1, and E-Selectin was induced mediating enhanced adhesion of leukocytes as an initial step of inflammation (Figure S8D–F). By contrast, TNF-α stimulation decreased eNOS mRNA expression, while Hsp70 protein expression was induced (Figure S8G,H). By analyzing Ea.hy926 hybrid EC, we have previously shown an upregulation of ROS at 24 h after 0.5 Gy X-ray exposure. In parallel, SOD1, GPx1, and Nrf2 expression and transcriptional activity showed a non-linear dose–response relationship with a local minimum at 0.5 Gy when cells were cultured for 30 min at 4 °C under non-laminar shear stress [51]. These data differ from the findings of the present study with an increase at a 0.1 Gy exposure, they may, however, arise from the former usage of hybrid cells, derived from the fusion of a tumor cell line (A549 lung cancer) and human umbilical vein endothelial cells (HUVEC), and differences in culture conditions with non-laminar shear stress and a different temperature. Similarly, protein expression of GPx was slightly downregulated at 0.5 Gy, compared to 0.7 and 1 Gy irradiation in HMVEC cells, while 0.1 Gy irradiation was not analyzed in our former study [51]. Again, this discrepancy most probably arises from different culture conditions [20].

ROS signaling has to be strictly controlled to defend against the deleterious consequences of ROS and to restore a cellular redox homeostasis. Accordingly, the coordinated induction of cytoprotective genes by the presence of an ARE in their promoter regions is essential for cellular protection against oxidative stress [15,52]. Gene transcription is activated by binding of the transcription factor Nrf2 to this motif, which commonly regulates the transcription of anti-oxidant factors and ROS detoxifying enzymes, thus covering a major regulator of the anti-oxidative response. Furthermore, Nrf2 is also influenced by atheroprotective shear stress acting on endothelium [53]. As Nrf2 declines in the vasculature with age, this could be an explanation for the increase in chronic inflammatory diseases in elderly people [54]. Patients undergoing LD-RT are mostly of older age and suffering from chronic inflammatory musculoskeletal diseases [45]. Our findings that Nrf2 mRNA is upregulated after low doses of X-rays as well as C-ions (Figure 1B and Figure 4B) are in line with the reports of a reduction of typical inflammatory symptoms like pain or movement restrictions. We also found significant upregulation of anti-oxidant enzymes downstream of Nrf2 (SOD1, GPx1 and Catalase) as well as reduced ROS levels (Figure 1 and Figure 4), although changes were not very pronounced. This could be due to the time point chosen in the present study (24 h after irradiation).

In this study, we did not observe a modulation of PBL adhesion nor a modulation of anti-oxidative marker or adhesion molecule mRNA and protein expression after X-ray or C-ion irradiation when cells were incubated under conventional static conditions (Figure 2 and Figure 5). The importance of “hemodynamic forces” acting on endothelium is well established [17]. Hence, a physiological quantity of laminar shear stress is essential for endothelial cells to maintain crucial functions and to integrate inflammatory stimuli [22]. Physiological flow further upregulates genes coding for anti-oxidant enzymes [43,54] mediated by Nrf2, while NAC abolishes this effect underlining the importance of a balanced ROS signaling [43]. Since sustained vascular dysfunction contributes to a variety of diseases including atherosclerosis [9], the efficiency of antioxidants in restoring the endothelial cell function has been widely investigated under different conditions in vitro and in vivo. For instance, treatment of HUVEC with NAC, ascorbic acid or propionyl-L-carnitine was sufficient to prevent TNF-α induced cell adhesion molecule expression, NADPH Oxidase 4 (Nox 4) expression and leukocyte adhesion [55]. A further endorsement of the interrelationship between ROS and endothelial cell function was reported by Deem et al. [56]. Treatment of murine mHEVc endothelial cells with 1 µM H_2_O_2_ activated matrix-metalloproteinase (MMP)-2 and MMP-9, a mechanism implicated in VCAM-1-dependent lymphocyte migration. By contrast, Catalase treatment prevented VCAM-1-mediated MMP activation, further providing a hint on the involvement of ROS in lymphocyte migration. These reports, along with our findings concerning an increased PBL adhesion (Figure 6) upon H_2_O_2_ treatment of HMVEC under laminar flow conditions, may confirm evidence for a pivotal correlation of inflammatory stimulation of the endothelium and ROS signaling. Yet, the detailed regulation of anti-oxidative enzymes following ROS induction and irradiation remains to be fully established and is most likely dependent on a multitude of factors, such as time course of the reaction, model system, or cultivation conditions.

Our findings may increase our knowledge of the effects underlying the clinical benefit of LD-RT with either photons or Radon. While there are no current data for possible long-term effects on EC function after LD-RT, much is known about the outcome after classical high-dose cancer therapy. Here, fractionated doses of up to 70 Gy are usually applied, and it is a well-known problem that radiation, e.g., for the treatment of breast cancer, can lead to cardiovascular diseases years after treatment [54], but the use of chemotherapeutic agents is often involved and cannot be well separated from radiation effects alone. In our in vitro setting, we analyzed doses, e.g., 0.5 Gy, typical for LD-RT and we simulated inflammation by adding TNF-α as most patients suffer from chronic inflammatory conditions. Accordingly, our data strengthen the role of an anti-inflammatory reaction in clinical therapeutic effects of LD-RT most likely involving a decrease in endothelial activation and leukocyte adhesion. However, here, we focused on doses used mostly in photon LD-RT. In Radon therapy, patients are exposed to even much lower doses (2 mSv, [33]). This is currently addressed in other studies.

## 5. Conclusions

**Figure 7:**
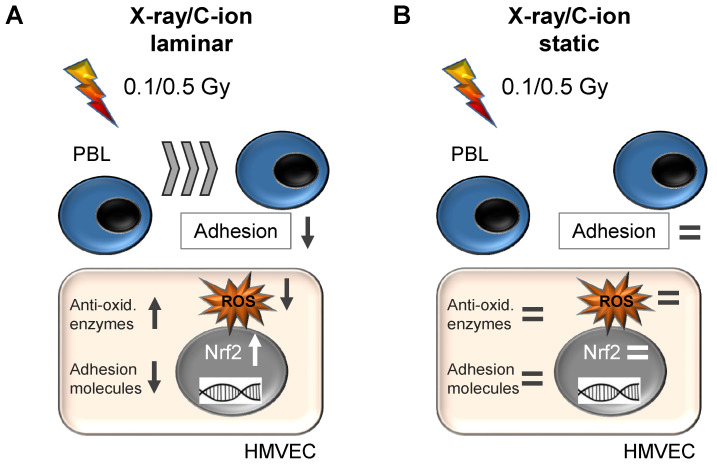
Irradiation of primary endothelial cells (HMVEC) with X-ray and C-ions induces shear stress-dependently the expression of anti-oxidative factors, reduces ROS and decreases PBL adhesion at doses between 0.1 and 0.5 Gy only under physiological shear stress. (**A**) X-ray or carbon ion (C-ion) irradiation with low doses induces under laminar conditions the expression of the transcription factor Nrf2, expression of anti-oxidative enzymes (Anti-ox. enzymes), decreases adhesion molecule expression and PBL adhesion, thereby probably contributing to anti-inflammatory effects of low-dose radiation therapy. (**B**) In contrast, low-dose irradiation of HMVEC cells under normal, static cell culture conditions does not modulate the anti-oxidative system or leukocyte adhesion.

In summary, our experimental data support the preclinical hypothesis that irradiation with single doses between 0.1 and 0.5 Gy of both X-rays and C-ions seems to be most effective for inducing anti-inflammatory responses in endothelial cells. In line with that, here, we propose a relationship of irradiation of endothelial cells, oxidative stress defense, and PBL adhesion. Under physiological laminar shear stress, the irradiation of HMVEC induces the mRNA expression of Nrf2 and anti-oxidative enzymes, resulting in a decreased metabolic ROS content, contributing to the reduced expression of adhesion molecules and decreased PBL adhesion and to anti-inflammatory effects of low-dose irradiation (Figure 7A). On the other hand, the irradiation of HMVEC, while cultivated under non-physiological static cell culture conditions, does not yield an anti-inflammatory response of immune cells (Figure 7B). Different LET have an impact on the local minimum in the non-linear dose–response relationship, which is for ROS at 0.1 Gy for X-rays and at 0.5 Gy for higher LET C-ions, contributing to a decreased PBL adhesion at 0.1 and 0.5 Gy. In conclusion, we suggest similar mechanisms underlying the anti-inflammatory effects of photon or Radon LD-RT, both involving the anti-oxidative system of endothelial cells.

## Figures and Tables

**Table 1:** Mean number of hits per nucleus after irradiation with Carbon (C)-ions. Calculation of mean number of hits per nucleus (nuclear area of human microvascular endothelial cells (HMVEC) = 200 µm^2^) and number of cells with 0 hit after irradiation with C-ions (linear energy transfer (LET_H2O_) = 323 keV/µm) according to the Poisson distribution.

Dose (Gy)	*Fluence* (Particles/cm^2^)	Mean Number of Hits/Nucleus *N*	Cells with 0 Hit *P*_0_
0.1	1.92 × 10^5^	0.38	0.68060781
0.5	9.60 × 10^5^	1.92	0.14604431
1	1.92 × 10^6^	3.85	0.02132894
2	3.84 × 10^6^	7.70	0.00045492

## Data Availability

All the data generated and/or analyzed in this study has been included in this article.

## Supplementary Materials

The following are available online at https://www.mdpi.com/article/10.3390/cells11010072/s1, Figure S1: Low-dose X-ray irradiation reduces reactive oxygen species (ROS) and leukocyte adhesion to human microvascular endothelial cells (HMVEC) under laminar conditions but not under static conditions, Figure S2: mRNA expression of eNOS and heat shock protein 70 (Hsp70) protein expression are not modulated by X-ray irradiation, Figure S3: Low-dose photon irradiation under laminar conditions does not enhance DNA damage or apoptosis, Figure S4: Low-dose carbon (C)-ion irradiation reduces ROS and leukocyte adhesion to HMVEC under laminar conditions but not under static conditions, Figure S5: mRNA expression of eNOS is not modulated by C-ion irradiation with doses up to 1 Gy, Figure S6: C-ion low-dose irradiation does not enhance apoptosis, Figure S7: Oxidative stress induces leukocyte adhesion to endothelial cells under static cell culture conditions, Figure S8: TNF-α treatment modulates ROS, expression of anti-oxidative factors and adhesion molecules, eNOS and Hsp70 protein expression and PBL adhesion, Table S1: Primer and probes for Real-Time PCR, Table S2: TaqMan Gene Expression Assays for Real-Time PCR.
