# Supplementary material for: ROS- and Radiation Source-Dependent Modulation of Leukocyte Adhesion to Primary Microvascular Endothelial Cells
Source: Cells. 2021 Dec 27;11(1):72. doi: 10.3390/cells11010072 (PMC8750044; doi:10.3390/cells11010072)
Supplement: Supplementary file 1 [file cells-11-00072-s001.zip › cells-1352097-supplementary.pdf]

ROS- and Radiation Source-Dependent Modulation of Leukocyte Adhesion to Primary Microvascular Endothelial Cells

Denise Eckert [1,#], Felicitas Rapp [1,#], Ayele T. Tsedeke [1], Jessica Molendowska [1], Robert Lehn [2], Markus Langhans [3], Claudia Fournier [1], Franz Rödel [4] and Stephanie Hehlgans [4,*]

Supplemental Figures

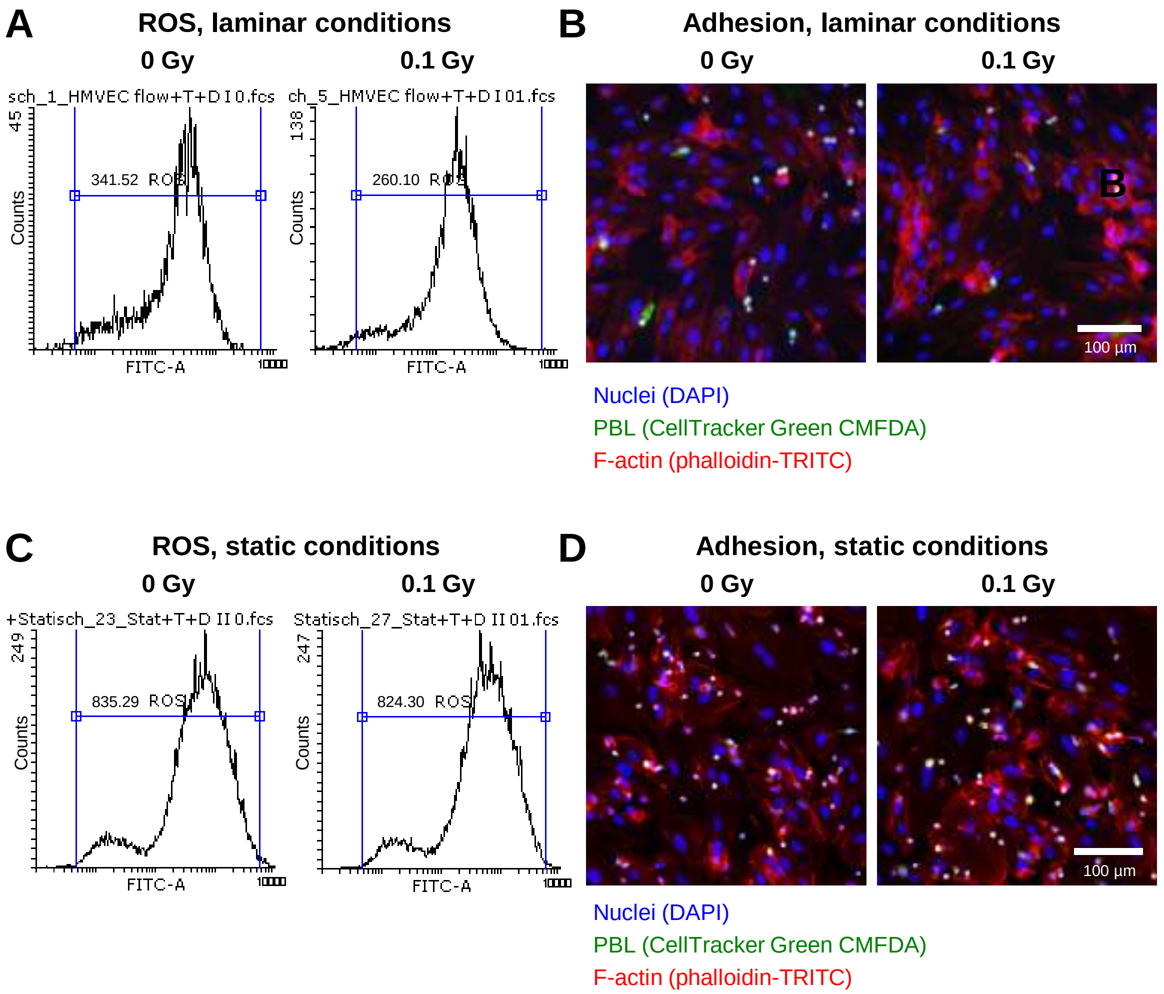

Figure S1. Low-dose X-ray irradiation reduces reactive oxygen species (ROS) and leukocyte adhesion to human microvascular endothelial cells (HMVEC) under laminar conditions but not under static conditions. ROS were measured 24 h after X-ray irradiation and tumor necrosis factor alpha (TNF-α) treatment under laminar conditions (**A**) or static conditions (**C**) by flow cytometry. Exemplary pictures are shown for indicated doses. For analysis of peripheral blood lymphocyte (PBL) adhesion under laminar conditions (**B**) or static conditions (**D**), HMVEC cells and adhered PBL, stained with CellTracker Green CMFDA (5-chloromethylfluorescein diacetate) before the assay was fixed and stained with DAPI (blue) and phalloidin-tetramethylrhodamine B isothiocyanate (phalloidin-TRITC, red) to visualize F-actin. Exemplary photographs of indicated conditions are shown. Bar, 100 µm.

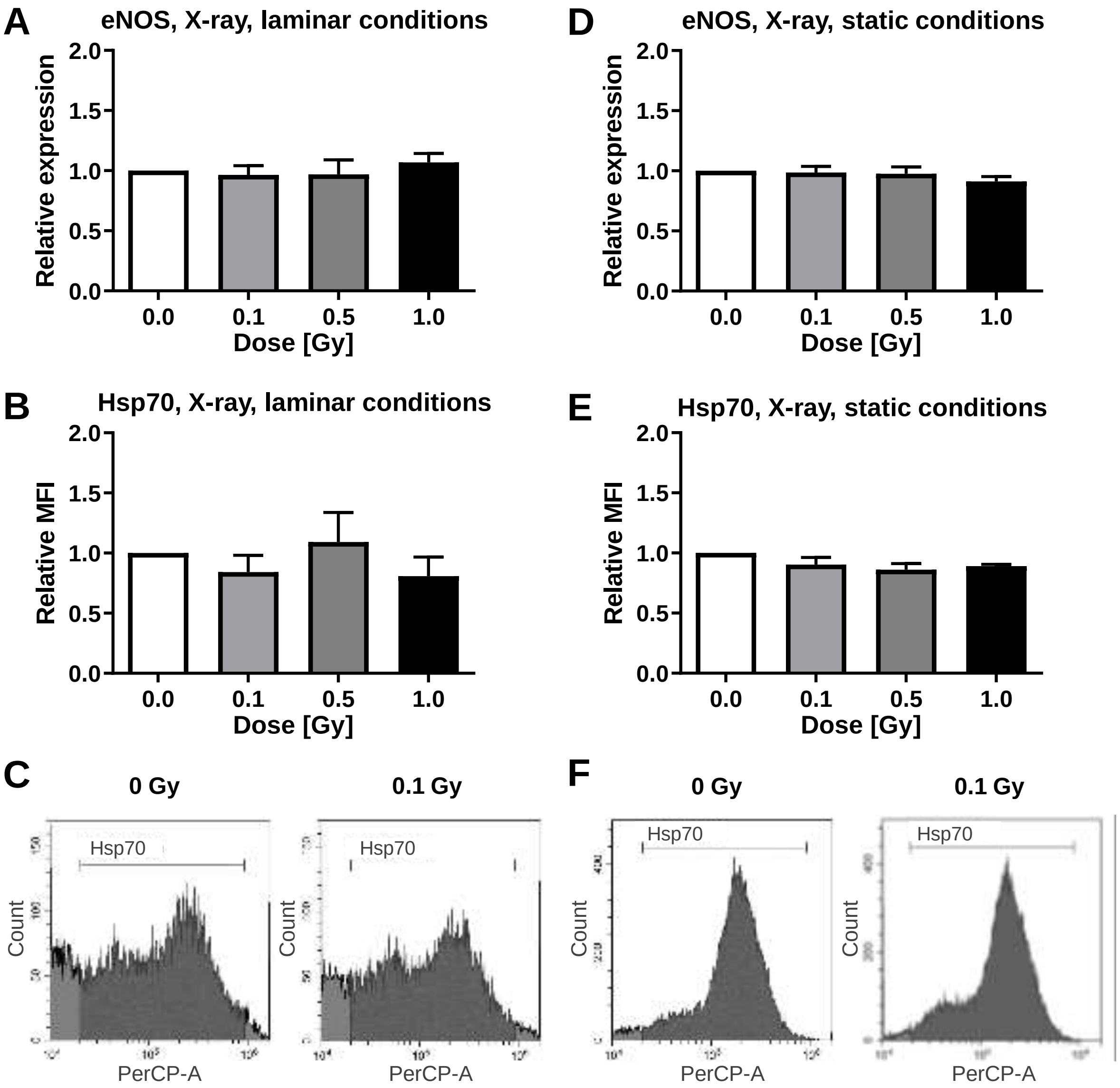

Figure S2. mRNA expression of eNOS and heat shock protein 70 (Hsp70) protein detection are not modulated by X-ray irradiation. (**A**) Expression of endothelial nitric oxide synthase (eNOS, gene name: *NOS3*) mRNA in HMVEC after X-ray was measured under laminar conditions by quantitative PCR (qPCR). Mean values + SEM relative to non-irradiated cells are shown (n = 6). (**B**) Hsp70 protein expression in HMVEC cells, irradiated with X-rays under laminar shear stress was evaluated by flow cytometry. Mean fluorescence intensities (MFI) + SEM, relative to non-irradiated cells are shown (n = 4). (**C**) Exemplary pictures of flow cytometric Hsp70 measurements are shown for indicated doses. (**D**) Expression of eNOS mRNA was evaluated after X-ray irradiation in HMVEC cultured under static conditions by qPCR (mean + SEM; n = 6). (**E**) Hsp70 protein expression in HMVEC cells, irradiated with X-rays under normal static conditions, was evaluated by flow cytometry (MFI + SEM; n = 4). (**F**) Exemplary pictures of measurements under static conditions are shown.

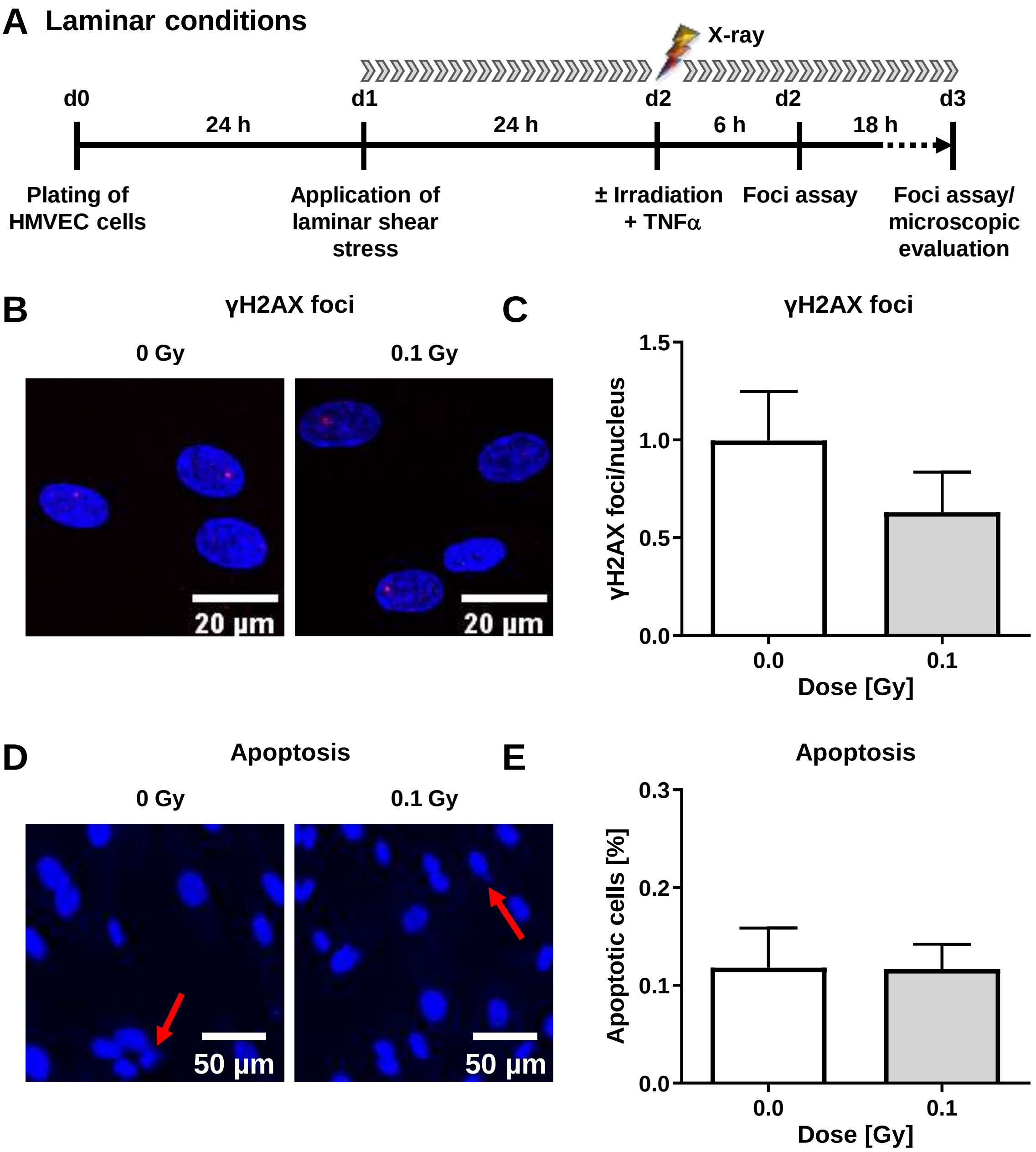

Figure S3. Low-dose photon irradiation under laminar conditions does not enhance DNA damage or apoptosis. The experimental time course of DNA damage and apoptosis detection under laminar conditions is depicted in (**A**). (**B**) DNA double-strand breaks (DBS) were quantified by immunofluorescence staining for phospho-Histone 2AX Ser139 (γH2AX, red channel), counterstaining with DAPI (blue channel) and microscopic counting of nuclear foci. (**C**) The mean number of γH2AX foci per nucleus + SEM is shown (n = 4). (**D**) Percentage of apoptosis was measured by counting of nuclei (DAPI, blue channel) with typical apoptotic morphology from laminar conditions relative to all nuclei in the respective microscopic field. Exemplary pictures are shown with red arrows depicting apoptotic bodies. (**E**) Percentage of apoptosis is shown for indicated conditions (mean + SEM; n = 3).

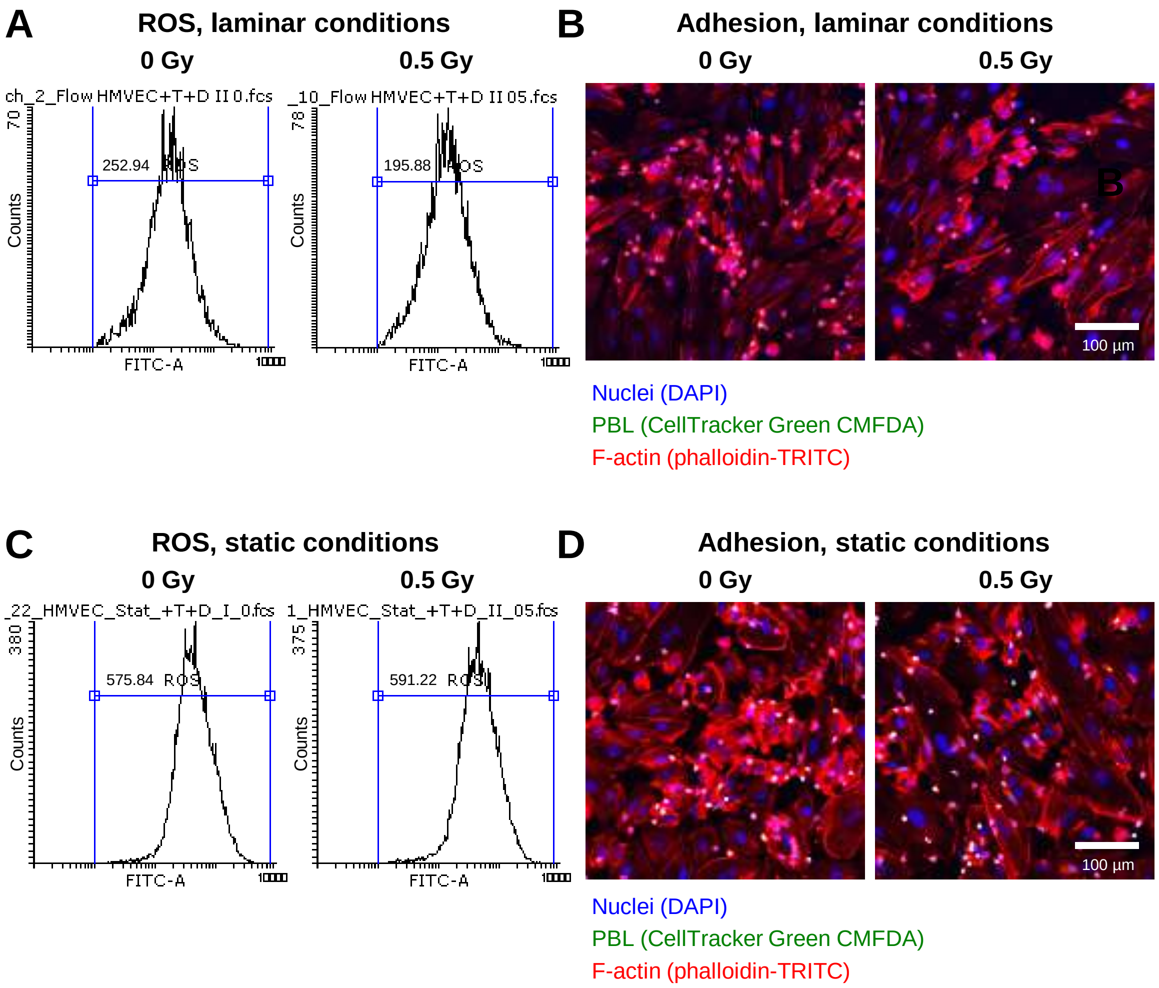

Figure S4. Low-dose carbon (C)-ion irradiation reduces ROS and leukocyte adhesion to HMVEC under laminar conditions but not under static conditions. ROS were measured 24 h after C-ion irradiation and TNF-α treatment under laminar conditions (**A**) or static conditions (**C**) by flow cytometry. Exemplary pictures are shown for indicated doses. For analysis of PBL adhesion under laminar conditions (**B**) or static conditions (**D**), HMVEC cells and adhered PBL, stained with CellTracker Green CMFDA before application to HMVEC, were fixed and stained with DAPI (blue) and phalloidin-TRITC (red) to visualize F-actin. Exemplary photographs of indicated conditions are shown. Bar, 100 µm.

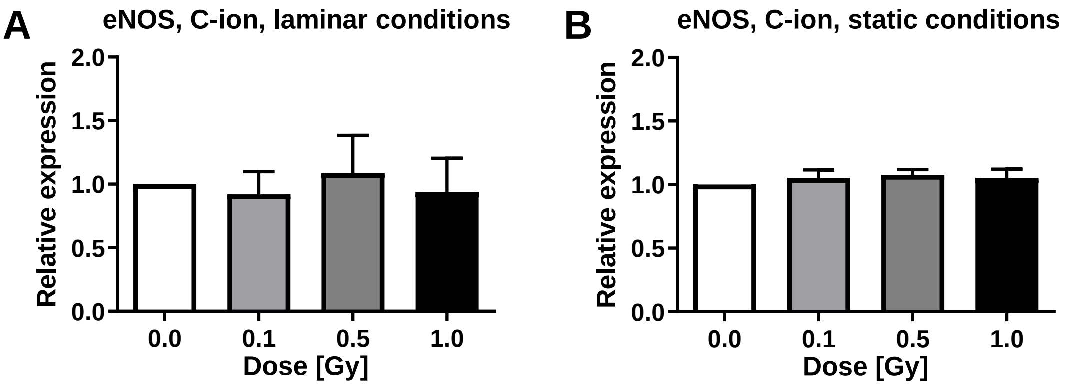

Figure S5. mRNA expression of eNOS is not modulated by C-ion irradiation with doses up to 1 Gy. Expression of eNOS mRNA after C-ion irradiation of shear stress-exposed HMVEC cells (**A**) or HMVEC cells cultured under normal conditions (**B**) was analyzed by qPCR. Mean values + SEM relative to non-irradiated cells are shown. n = 4 (laminar) or n = 8 (static).

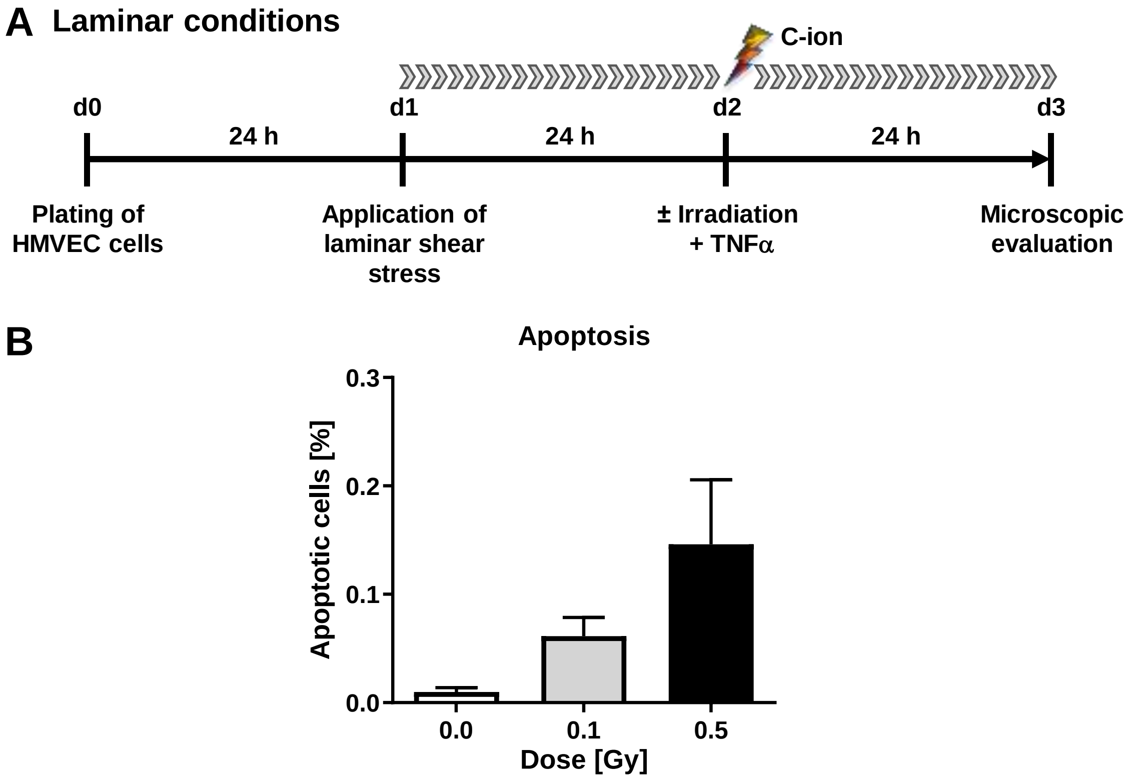

Figure S6. C-ion low-dose irradiation does not enhance apoptosis. (**A**) For measurement of apoptosis, HMVEC were exposed to laminar flow conditions, irradiated with C-ions and TNF-α stimulated. (**B**) At 24 h after irradiation, cells were fixed and stained with DAPI, followed by microscopic evaluation of nuclei with typical apoptotic morphology. The percentage of apoptosis at indicated doses is displayed (mean + SEM; n = 3).

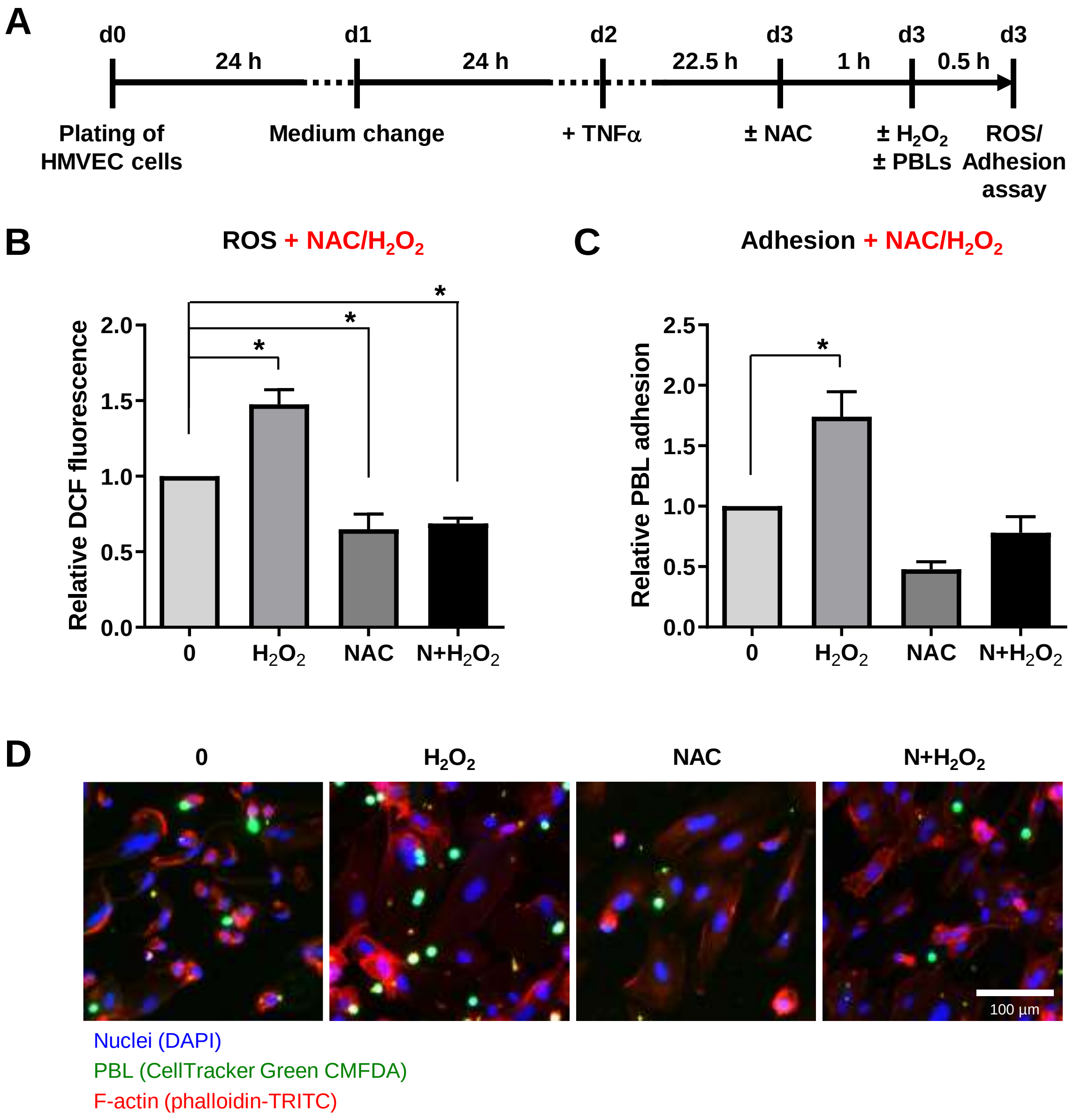

Figure S7. Oxidative stress induces leukocyte adhesion to endothelial cells under static cell culture conditions. (**A**) Experimental scheme: HMVEC were plated and after 24 h treated with TNF-α. After 22.5 h, cells were either treated with the ROS scavenger N-acetyl-L-cysteine (NAC) or left untreated. Hydrogen peroxide (H_2O_2) was applied to induce oxidative stress either without or with pretreatment with NAC (N+H_2O_2). (**B**) ROS were measured by flow cytometry. (**C**) In case of adhesion experiments, Cell Tracker Green-stained PBL were added to the cell culture dishes with HMVEC cells and adhesion to HMVEC was allowed for 30 min. Mean values + SEM relative to untreated cells (0) are shown. n = 5-6; * $p < 0.05$ (one-way ANOVA vs. untreated (0)). (**D**) Exemplary pictures, depicting adhered PBL (CellTracker Green CMFDA), nuclei (DAPI, blue) and F-actin staining (phalloidin-TRITC, red) at indicated conditions. Bar, 100 µm.

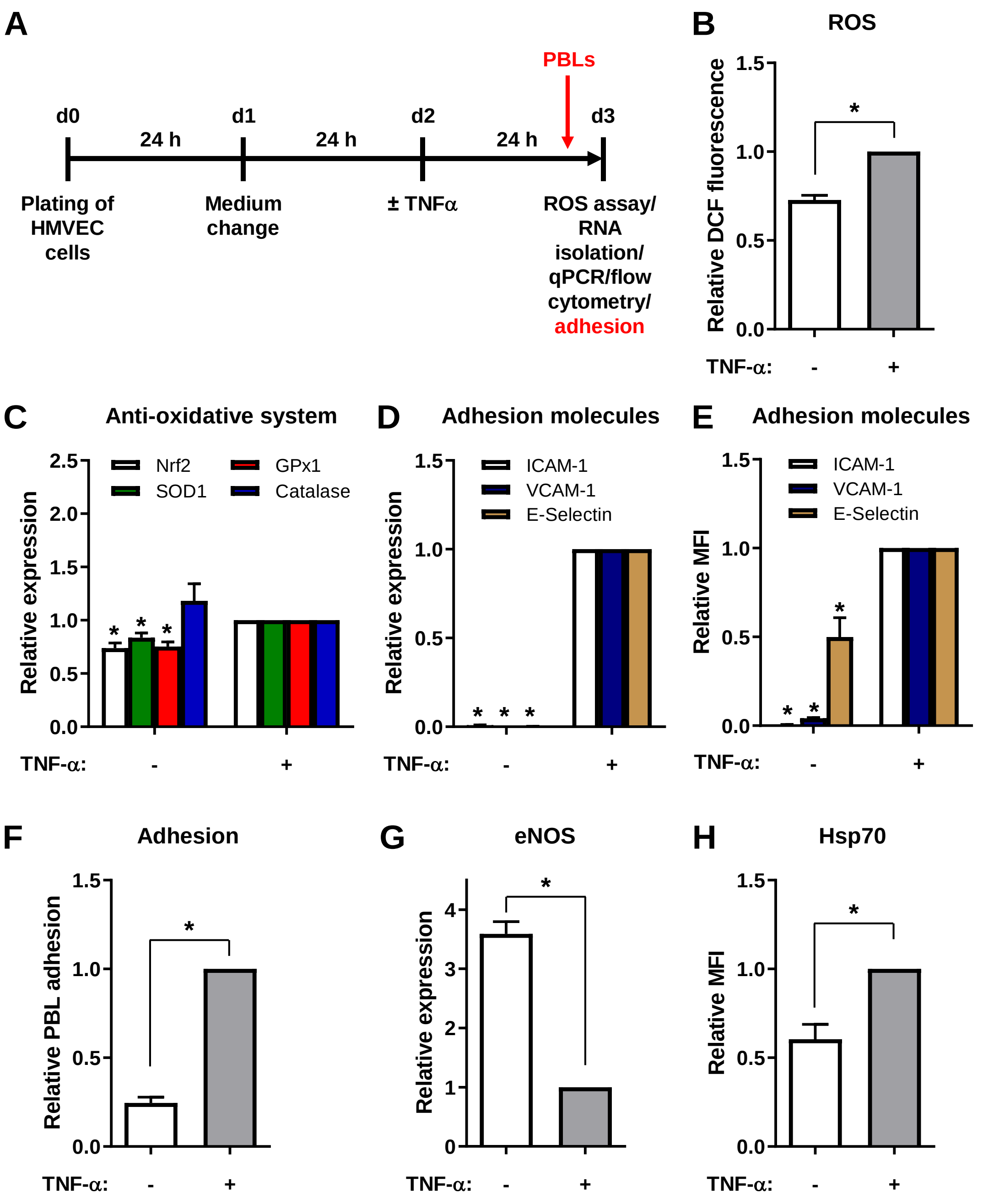

Figure S8. TNF-α treatment modulates ROS, expression of anti-oxidative factors, adhesion molecules, eNOS and Hsp70 protein expression and PBL adhesion. (**A**) For all static experiments, non-irradiated control cells without TNF-α treatment were compared to TNF-α treated non-irradiated cells. HMVEC were plated and incubated for 24 h. Medium was changed and after 24 h, cells were treated with TNF-α (+TNF-α) or left untreated (-TNF-α). After 24 h, cells were subjected either to ROS measurement, RNA isolation for evaluation of expression of anti-oxidative factors (Nrf2 (Nuclear factor-erythroid-2-related factor 2), SOD1 (Superoxide Dismutase 1), GPx1 (Glutathione Peroxidase 1), Catalase) and adhesion molecules (ICAM-1 (intercellular adhesion molecule 1), VCAM-1 (vascular cell adhesion molecule 1), E-Selectin) or adhesion assays. For adhesion assays, PBL were added 30 min before analysis. (**B**) Fluorescence of DCF-treated HMVEC was measured by flow cytometry. Mean values + SEM relative to TNF-α stimulated cells are shown. n = 26; * $p < 0.05$ (Mann-Whitney U test vs. +TNF-α). (**C**) Relative expression of anti-oxidative factors and (**D**) adhesion molecules was evaluated by quantitative PCR. Mean + SEM; n = 12-14; * $p < 0.05$ (Mann-Whitney U test vs. +TNF-α). (**E**) Mean fluorescence intensities (MFI) for adhesion molecules are shown relative to TNF-α stimulated HMVEC, measured by flow cytometric analysis. n = 4; * $p < 0.05$ (Mann-Whitney U test vs. +TNF-α). (**F**) Relative PBL adhesion to non-treated vs. TNF-α treated HMVEC is shown. n = 19; * $p < 0.05$ (Mann-Whitney U test vs. +TNF-α). (**G**) Relative expression of eNOS was measured by qPCR. n = 6; * $p < 0.05$ (Mann-Whitney U test vs. +TNF-α). (**H**) Hsp70 protein expression was measured by flow cytometry and Mean fluorescence intensities (MFI) are shown relative to TNF-α stimulated HMVEC. n = 4; * $p < 0.05$ (Mann-Whitney U test vs. +TNF-α).

Supplemental Tables

Table S1. Primer and probes for Real-Time PCR.

Gene	Sequence (5′→3′)	Reference
**hSOD1 (forward)**	GGTCCTCACTTTAATCCTCTATCCAG	[1,2]
**hSOD1 (reverse)**	CCAACATGCCTCTCTTCATCC	
**hSOD1 (probe)**	[FAM]AACACGGTGGGCCAA[TAMRA]	
**hGPx1 (forward)**	TGTGCCCCTACGCAGGTACA	[3]
**hGPx1 (reverse)**	CCCCCGAGACAGCAGCA	
**hGPx1 (probe)**	[FAM]CTGTCTCAAGGGCCCAGCTGTGC[TAMRA]	
**hCatalase (forward)**	TTAATCCATTCGATCTCACC	[4]
**hCatalase (reverse)**	GGCGGTGAGTGTCAGGATAG	
**hCatalase (probe)**	[FAM]AGGCTATCTGTTCAACCTCAGCAAAGTAAT[TAMRA]	
**hRPL37A (forward)**	TGTGGTTCCTGCATGAAGACA	[2,5]
**hRPL37A (reverse)**	GTGACAGCGGAAGTGGTATTGTAC	
**hRPL37A (probe)**	[FAM]TGGCTGGCGGTGCCTGGA[TAMRA]	

Primer and probes for Real-Time PCR were manufactured at Eurofins Genomics, Ebersberg, Germany. Abbreviations: FAM, 6-carboxyfluorescein; hCatalase, homo sapiens Catalase; hGPx1, homo sapiens glutathione peroxidase; hRPL37A, homo sapiens 60S ribosomal protein L37a; hSOD1, homo sapiens superoxide dismutase 1; TAMRA, tetramethylrhodamine.

References

1. Gagliardi, S.; Cova, E.; Davin, A.; Guareschi, S.; Abel, K.; Alvisi, E.; Laforenza, U.; Ghidoni, R.; Cashman, J.R.; Ceroni, M., et al. SOD1 mRNA expression in sporadic amyotrophic lateral sclerosis. *Neurobiol Dis* **2010**, *39*, 198-203.
2. Large, M.; Hehlgans, S.; Reichert, S.; Gaipl, U.S.; Fournier, C.; Rodel, C.; Weiss, C.; Rodel, F. Study of the anti-inflammatory effects of low-dose radiation: The contribution of biphasic regulation of the antioxidative system in endothelial cells. *Strahlenther Onkol* **2015**, *191*, 742-749.
3. Ratnasinghe, D.; Tangrea, J.A.; Andersen, M.R.; Barrett, M.J.; Virtamo, J.; Taylor, P.R.; Albanes, D. Glutathione peroxidase codon 198 polymorphism variant increases lung cancer risk. *Cancer Res* **2000**, *60*, 6381-6383.
4. Hasegawa, Y.; Takano, T.; Miyauchi, A.; Matsuzuka, F.; Yoshida, H.; Kuma, K.; Amino, N. Decreased expression of catalase mRNA in thyroid anaplastic carcinoma. *Jpn J Clin Oncol* **2003**, *33*, 6-9.
5. Muller, B.M.; Kronenwett, R.; Hennig, G.; Euting, H.; Weber, K.; Bohmann, K.; Weichert, W.; Altmann, G.; Roth, C.; Winzer, K.J., et al. Quantitative determination of estrogen receptor, progesterone receptor, and HER2 mRNA in formalin-fixed paraffin-embedded tissue--a new option for predictive biomarker assessment in breast cancer. *Diagn Mol Pathol* **2011**, *20*, 1-10.

Table S2. TaqMan Gene Expression Assays for Real-Time PCR.

Gene	TaqMan Assay ID	Labeling
hNFE2L2	Hs00232352_m1	5′-FAM, 3′-MGB
**hICAM1**	Hs00164932_m1	5′-FAM, 3′-MGB
**hVCAM1**	Hs01003372_m1	5′-FAM, 3′-MGB
hSELE	Hs00174057_m1	5′-FAM, 3′-MGB
hNOS3	Hs01574659_m1	5′-FAM, 3′-MGB

TaqMan Gene Expression Assays were purchased from Thermo Fisher Scientific, Darmstadt, Germany. Abbreviations: FAM, 6-carboxyfluorescein; hICAM1, homo sapiens intercellular adhesion molecule 1; *hNFE2L2*, homo sapiens nuclear factor erythroid-derived 2-like 2 (gene encoding for human Nuclear factor erythroid 2-related factor 2 (hNrf2)); *hNOS3*, homo sapiens nitric oxide synthase 3 (gene encoding for endothelial nitric oxide synthase (eNOS)); *hSELE*, homo sapiens Selectin E; hVCAM1, homo sapiens vascular cell adhesion molecule 1; MGB, minor groove binder.
